# Supplementary material for: mRNA vaccine expressing enterovirus D68 virus-like particles induces potent neutralizing antibodies and protects against infection
Source: Mol Ther Nucleic Acids. 2025 Oct 6;36(4):102731. doi: 10.1016/j.omtn.2025.102731 (PMC12593654; doi:10.1016/j.omtn.2025.102731)
Supplement: Document S1. Figures S1–S8 and Table S1 [file mmc1.pdf]

OMTN, Volume *36*

Supplemental information

mRNA vaccine expressing enterovirus D68 virus-like particles induces potent neutralizing antibodies and protects against infection

Yuta Kunishima, Kota Senpuku, Chikako Kataoka-Nakamura, Toshiro Hirai, and Yasuo Yoshioka

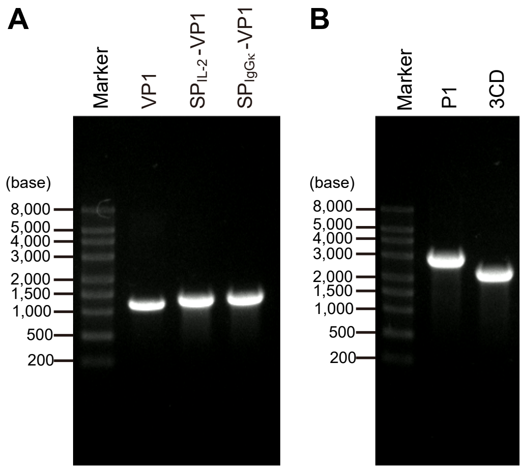

Figure S1. Electrophoresis of mRNA. (A, B) The denatured mRNA samples were electrophoresed in a 1% agarose gel. Marker shows single-stranded RNA ladder. (A) The mRNAs used in Figure 1. (B) The mRNAs used in Figures 2–6.

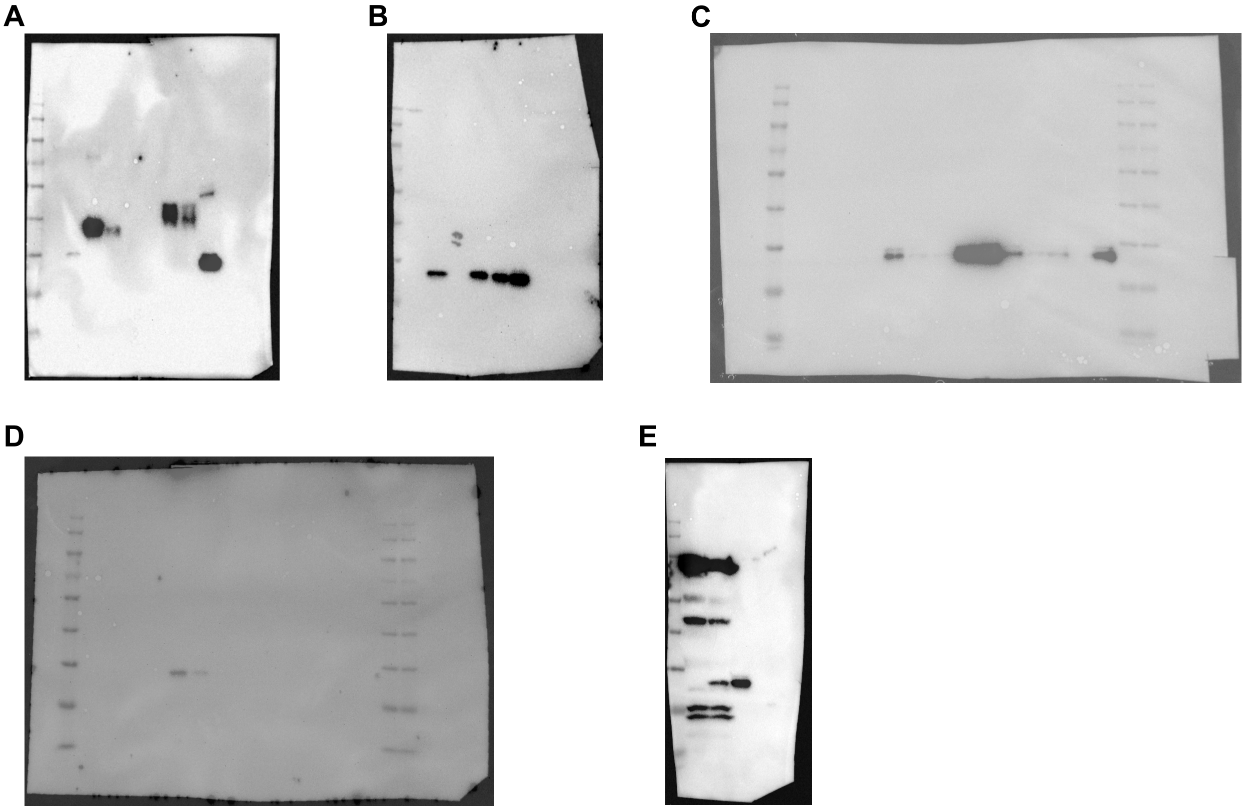

Figure S2. Unedited blotting images. (A) Figure 1B. (B) Figure 2B. (C,D) Figure 2C (E) Figure 2D.

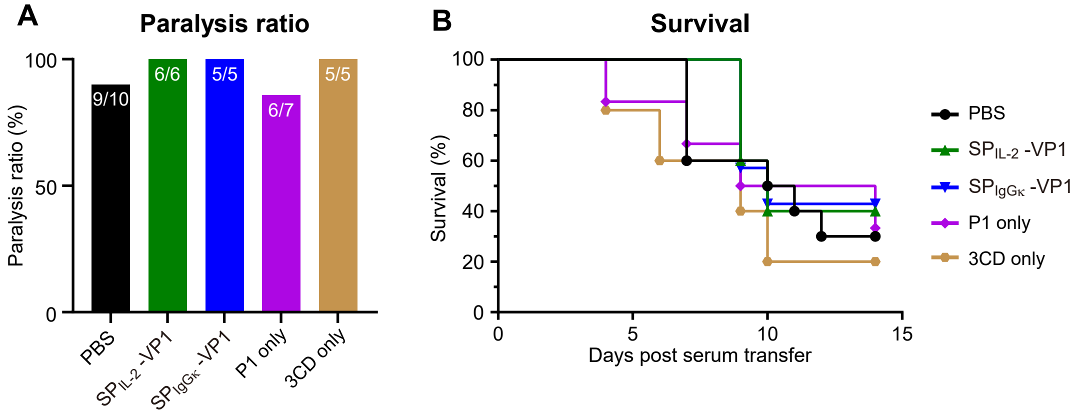

Figure S3. The protective effect of the sera derived from mice vaccinated for VP1, P1, or 3CD. Limb paralysis (A) and rate of survival (B) in the challenged mice were monitored every day for 14 days after the serum transfer. Experiment schema is shown in Figure 3C.

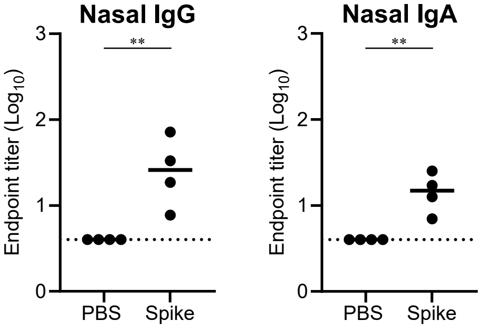

Figure S4. Mucosal antibodies induced by intramuscular vaccination of SARS-CoV-2 mRNA vaccine. Anti-Spike IgG and IgA in nasal wash induced by twice intramuscular immunization of SARS-CoV-2 Spike mRNA-LNP (total 2 μg as mRNA per dose) were quantified with ELISA. Points are presented as individual data and lines are presented as the median. Dotted line represents the detection limit. **, $P < 0.01$ as indicated by Student's *t*-test.

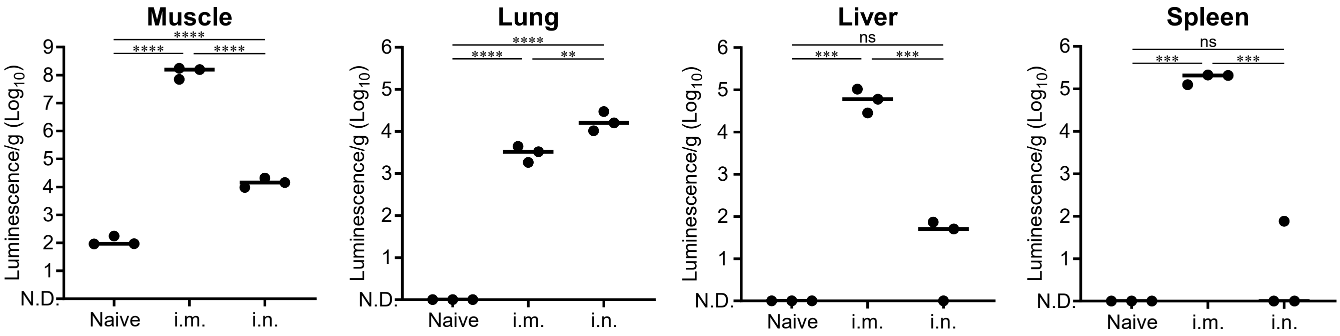

Figure S5. Tissue distribution and expression of mRNA-LNP administrated intramuscularly or intranasally. The mRNA encoding firefly luciferase was encapsulated into LNP and administrated intramuscularly (i.m.) or intranasally (i.n.) to mice (3 μg RNA per dose). The muscle, lung, liver, and spleen were harvested 6 h after administration, and the luciferase activity in the tissue homogenate was quantified. The luminescence was standardized by tissue weight. Points are presented as individual data and lines are presented as median. N.D. means not detectable. **, $P < 0.01$; ***, $P < 0.001$; ****, $P < 0.0001$; ns, not significant as indicated by Tukey's test. (Continued from Fig. 5G.)

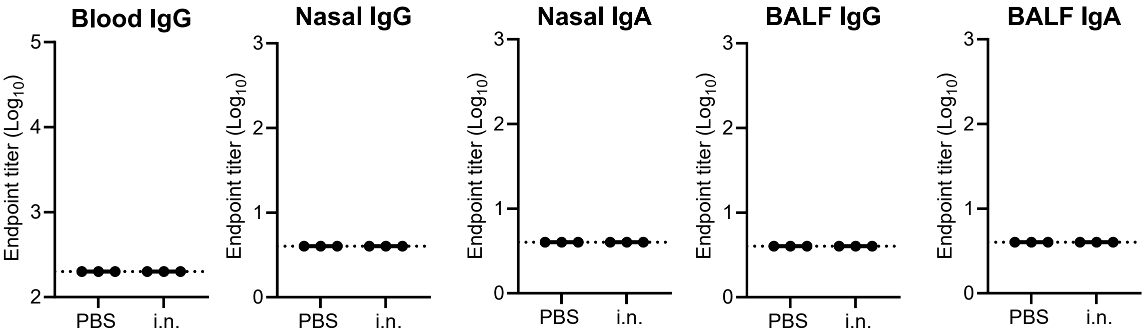

Figure S6. Systemic and mucosal antibodies induced by intranasal vaccination of the mRNA vaccine expressing VLP. Anti-EV-D68 IgG in plasma, IgG and IgA in nasal wash, and IgG and IgA in BALF induced by twice intranasal vaccination of the mRNA vaccine expressing VLP (P1:3CD = 1:1 on weight, total 2 μg as mRNA per dose) were quantified with ELISA. Points are presented as individual data and lines are presented as the median. Dotted line represents the detection limit.

```
EV-D68_MO_3CD      GPGFDFAQAIMKKNTVIARTEKGEFTMLGVYDRVAVIPTHASVGETIYINDVETKVLDAC
CVB3_Nancy_3CD     GPAFEFAVAMMKRNSSTVKTEYGEFTMLGIYDRWAVLPRHAKPGPTILMNDQEVGVLDAK
                   ** * ** * ** *     ** ******* *** ** * **  * **  ** *  **** 

EV-D68_MO_3CD      ALRDLTDTNLEITIVKLDRNQKFRDIRHFLPRYEDDYNDAVLSVHTSKFPNMYIPVGQVT
CVB3_Nancy_3CD     ELVDKDGTNLELTLLKLNRNEKFRDIRGFLAKEEVEVNEAVLAINTSKFPNMYIPVGQVT
                    * *   ***  * *  ** ** ****** **   *   * ***   ***************

EV-D68_MO_3CD      NYGFLNLGGTPTHRILMYNFPTRAGQCGGVVTTTGKVIGIHVGGNGAQGFAAMLLHSYFS
CVB3_Nancy_3CD     EYGFLNLGGTPTKRMLMYNFPTRAGQCGGVLMSTGKVLGIHVGGNGHQGFSAALLKHYFN
                    *********** * ***************   **** ******** *** * **  ** 

EV-D68_MO_3CD      DTQGEIVSSEKSG----VCINAPAKTKLQPSVFHQVFEGSKEPAVLNPKDPRLKTDFEEA
CVB3_Nancy_3CD     DEQGEIEFIESSKDAGFPVINTPSKTKLEPSVFHQVFEGNKEPAVLRSGDPRLKANFEEA
                   * ****   * *       ** * **** ********* ******   *****  ****

EV-D68_MO_3CD      IFSKYTGNKIMLMDEYMEEAVDHYVGCLEPLDISVDPIPLESAMYGMDGLEALDLTTSAG
CVB3_Nancy_3CD     IFSKYIGNVNTHVDEYMLEAVDHYAGQLATLDISTEPMKLEDAVYGTEGLEALDLTTSAG
                   ***** **     **** ****** * *  ****  *  ** * **  ************

EV-D68_MO_3CD      FPYLLQGKKKRDIFNRHTRDTSEMTKMLEKYGVDLPFVTFVKDELRSREKVEKGKSRLIE
CVB3_Nancy_3CD     YPYVALGIKKRDILSKKTKDLTKLKECMDKYGLNLPMVTYVKDELRSIEKVAKGKSRLIE
                    **   * *****    * *         ***  ** ** ******* *** ********

EV-D68_MO_3CD      ASSLNDSVAMRVAFGNLYATFHNNPGTATGSAVGCDPDIFWSKIPILLDGEIFAFDYTGY
CVB3_Nancy_3CD     ASSLNDSVAMRQTFGNLYKTFHLNPGVVTGSAVGCDPDLFWSKIPVMLDGHLIAFDYSGY
                   ***********  ***** *** ***  ********** ******  ***   **** **

EV-D68_MO_3CD      DASLSPVWFACLKKVLIKLGYTHQTS-FIDYLCHSVHLYKDKKYIVNGGMPSGSSGTSIF
CVB3_Nancy_3CD     DASLSPVWFACLKMLLEKLGYTHKETNYIDYLCNSHHLYRDKHYFVRGGMPSGCSGTSIF
                   *************  * ******     ***** * *** ** * * ****** ******

EV-D68_MO_3CD      NTMINNIIIRTLLIRVYKGIDLDQFKMIAYGDDVIASYPHKIDPGLLAEAGKQYGLVMTP
CVB3_Nancy_3CD     NSMINNIIIRTLMLKVYKGIDLDQFRMIAYGDDVIASYPWPIDASLLAEAGKGYGLIMTP
                   * **********   ********** *************  **  ******* *** ***

EV-D68_MO_3CD      ADKGTSFIDTNWENVTFLKRYFRADDQYPFLIHPVMPMKEIHESIRWTKDPRNTQDHVRS
CVB3_Nancy_3CD     ADKGECFNEVTWTNATFLKRYFRADEQYPFLVHPVMPMKDIHESIRWTKDPKNTQDHVRS
                   ****  *     * * ********** **** * ******* *********** ********

EV-D68_MO_3CD      LCYLAWHNGEEAYNEFCRKIRSVPVGRALTLPAYSSLRRKWLDSF
CVB3_Nancy_3CD     LCLLAWHNGEHEYEEFIRKIRSVPVGRCLTLPAFSTLRRKWLDSF
                   ** *******  * ** ********** ***** * *********
```

The conserved class I epitope is printed in red on the page: VLNPKDPRLK in EV-D68_MO_3CD and VLRSGDPRLK in CVB3_Nancy_3CD, along with the conservation marks beneath it.

Figure S7. Protein alignment of the 3CD region of the EV-D68 vaccine strain (MO) with the CVB3 challenge strain (Nancy). Alignment was performed using clastal w. Red charactor shows conserved class I epitope discribed in Discussion section.

```
EV-D68_MO_P1    MGAQVTRQQTGTHENANIATNGSHITYNQINFYKDSYAASASKQDFSQDPSKFTEPVVEG
CVB3_Nancy_P1   MGAQVSTQKTGAHETRLNASGNSIIHYTNINYYKDAASNSANRQDFTQDPGKFTEPVKDI
                *****  * ** **    *   * * *  ** ***    **  *** *** ******

EV-D68_MO_P1    LKAGAPVLKSPSAEACGYSDRVLQLKLGNSAIVTQEAANYCCAYGEWPNYLPDHEAVAID
CVB3_Nancy_P1   MIKSLPALNSPTVEECGYSDRARSITLGNSTITTQECANVVVGYGVWPDYLKDSEATAED
                     * * **  * ******     **** * *** **    ** ** ** * ** * *

EV-D68_MO_P1    KPTQPETATDRFYTLKSVKWETGSTGWWWKLPDALNNIGMFGQNVQHHYLYRSGFLIHVQ
CVB3_Nancy_P1   QPTQPDVATCRFYTLDSVQWQKTSPGWWWKLPDALSNLGLFGQNMQYHYLGRTGYTVHVQ
                 ****  ** ***** ** *   * ********** * * **** * *** * *   ***

EV-D68_MO_P1    CNATKFHQGALLVVAIPEHQRGAHNTNTSPGFDDIMKGEEGGTFNHP-------------
CVB3_Nancy_P1   CNASKFHQGCLLVVCVPEAEMGCATLDNTPSSAELLGGDSAKEFADKPVASGSNKLVQRV
                *** ***** ****  **   *       *       *     *

EV-D68_MO_P1    -YVLDDGTSLACATIFPHQWINLRTNNSATIVLPWMNAAPMDFPLRHNQWTLAIIPVVPL
CVB3_Nancy_P1   VYNAGMGVGVGNLTIFPHQWINLRTNNSATIVMPYTNSVPMDNMFRHNNVTLMVIPFVPL
                 *    *      ******************* *  *  ***   ***  **  ** ***

EV-D68_MO_P1    GTR-TTSSMVPITVSIAPMCCEFNGLRHAITQGVPTYLLPGSGQFLTTDDHSSAPALPCF
CVB3_Nancy_P1   DYCPGSTTYVPITVTIAPMCAEYNGLRLAGHQGLPTMNTPGSCQFLTSDDFQSPSAMPQY
                         ***** ***** * **** *  ** **   *** **** **  *  * *

EV-D68_MO_P1    NPTPEMHIPGQVRNMLEVVQVESMMEINNTESAVG-MERLKVDISALTDVDQLLFNIPLD
CVB3_Nancy_P1   DVTPEMRIPGEVKNLMEIAEVDSVVPVQNVGEKVNSMEAYQIPVRSNEGSGTQVFGFPLQ
                  **** *** * *  *   * *     *    *  **                *  **

EV-D68_MO_P1    IQLDGPLRNTLVGNISRYYTHWSGSLEMTFMFCGSFMAAGKLILCYTPPGGSCPTTRETA
CVB3_Nancy_P1   PGYSSVFSRTLLGEILNYYTHWSGSIKLTFMFCGSAMATGKFLLAYSPPGAGAPTKRVDA
                         ** * *  ********   ******* ** **  * * ***   ** *  *

EV-D68_MO_P1    MLGTHIVWDFGLQSSVTLIIPWISGSHYRMFNNDAKSTNANVGYVTCFMQTNLIVPSESS
CVB3_Nancy_P1   MLGTHVIWDVGLQSSCVLCIPWISQTHYRYVASDEYTAGG---FITCWYQTNIVVPADAQ
                *****  ** *****  * *****  ***    *           **  ***  **

EV-D68_MO_P1    DTCSLIGFIAAKDDFSLRLMRDSPDIGQLDHLHAAEAAYQIESIIKTATDTVKSEINAEL
CVB3_Nancy_P1   SSCYIMCFVSACNDFSVRLLKDTPFISQQNFFQGPVEDAITAAIGRVADTVGTGPTNSEA
                  *    *  *  *** **  * * * *               *   *        * *

EV-D68_MO_P1    GVVPSLNAVETGATSNTEPEEAIQTRTVINQHGVSETLVENFLSRAALVSKRSFEYKDHT
CVB3_Nancy_P1   --IPALTAAETGHTSQVVPGDTMQTRHVKNYHSRSESTIENFLCRSACVYFTEYENS---
                   * * * *** **   *    *** * * *  **   **** * * *     *

EV-D68_MO_P1    SSTARADKNFFKWTINTRSFVQLRRKLELFTYLRFDAEITILTTVAVNGSGNNTYVGLPD
CVB3_Nancy_P1   -----GAKRYAEWVLTPRQAAQLRRKLEFFTYVRFDLELTFVITSTQQPSTTQNQD-AQI
                       *    *    *   ******* *** *** * *   *     *

EV-D68_MO_P1    LTLQAMFVPTGALTPEKQDSFHWQSGSNASVFFKISDPPARITIPFMCINSAYSVFYDGF
CVB3_Nancy_P1   LTHQIMYVPPGGPVPDKVDSYVWQTSTNPSVFWTEGNAPPRMSIPFLSIGNAYSNFYDGW
                ** * * ** *   * * **  **   * ***      * *  ***  *  *** ****

EV-D68_MO_P1    AGFEKNGLYGINPADTIGNLCVRIVNEHQPVGFTVTVRVYMKPKHIKAWAPRPPRTLPYM
CVB3_Nancy_P1   SEFSRNGVYGINTLNNMGTLYARHVNAGSTGPIKSTIRIYFKPKHVKAWIPRPPRLCQYE
                  *  ** ****     * *  * **         * * * **** *** *****   *

EV-D68_MO_P1    SIANANYKGKERAPNALSAIIGNRDSVKTMPHNIVNT
CVB3_Nancy_P1   KAKNVNFQP--------SGVTTTRQSITTMTNTGAF-
                   * *           *     * *  **
```

Figure S8. Protein alignment of the P1 region of the EV-D68 vaccine strain (MO) with the CVB3 challenge strain (Nancy). Alignment was performed using clastal w.

Table S1. The template DNA for *in vitro* transcription used in this study All genes were inserted into the TAKARA IVTpro vector. The sequence of the secretory signaling peptide is underlined.

Gene	Sequence
EV-D68 MO VP1	ATGCTGGATCACCTTCATGCAGCAGAAGCTGCCTACCAGATTGAGTCCATCATCAAGACAGCCACAGA<br>TACCGTGAAATCCGAGATAAACGCTGAACTGGGTGTTGTTCCATCCCTGAATGCAGTAGAAACAGGAG<br>CCACTTCCAATACCGAACCTGAAGAGGCCATTCAGACACGCACCGTGATTAACCAGCATGGCGTAAGC<br>GAAACTCTGGTCGAGAATTTCCTCTCTCGCGCTGCCTTGGTCAGCAAACGCAGCTTCGAATACAAGGA<br>TCACACGTCATCAACCGCTAGAGCGGACAAGAACTTCTTCAAGTGGACTATCAACACACGGTCCTTTGT<br>GCAACTGCGTAGGAAACTGGAGCTCTTCACGTACCTCAGATTTGACGCGGAGATTACCATTCTCACCA<br>CTGTGGCTGTTAACGGCAGTGGAAACAATACTTATGTCGGTCTGCCTGACCTGACCTTGCAGGCAATG<br>TTTGTACCGACAGGAGCCCTTACTCCCGAGAAACAGGACAGCTTTCATTGGCAGTCAGGCAGCAATGC<br>TAGTGTGTTCTTCAAGATCAGTGATCCACCAGCTCGGATTACCATCCCCTTCATGTGCATCAACTCTGC<br>CTATAGCGTTTTCTATGACGGCTTTGCCGGATTTGAGAAGAATGGGCTGTATGGGATTAACCCTGCTGA<br>CACAATTGGCAATCTGTGTGTGCGGATCGTGAATGAGCACCAACCAGTGGGGTTTACAGTGACGGTGC<br>GAGTGTACATGAAGCCCAAACACATCAAGGCATGGGCACCCAGACCTCCTAGGACTCTTCCGTACATG<br>AGCATAGCCAACGCGAACTACAAAGGGAAAGAGCGAGCCCCAAATGCACTGTCTGCCATAATCGGTAA<br>CAGGGATTCTGTCAAGACCATGCCCCACAATATCGTCAACACCTGA
EV-D68 MO VP1<br>with human IL-2<br>secretory signal peptide	ATGTACAGAATGCAGCTCCTGTCCTGCATCGCTCTGTCTCTTGCCCTGGTGACCAACAGCCTGGATCA<br>CCTTCATGCAGCAGAAGCTGCCTACCAGATTGAGTCCATCATCAAGACAGCCACAGATACCGTGAAAT<br>CCGAGATAAACGCTGAACTGGGTGTTGTTCCATCCCTGAATGCAGTAGAAACAGGAGCCACTTCCAAT<br>ACCGAACCTGAAGAGGCCATTCAGACACGCACCGTGATTAACCAGCATGGCGTAAGCGAAACTCTGGT<br>CGAGAATTTCCTCTCTCGCGCTGCCTTGGTCAGCAAACGCAGCTTCGAATACAAGGATCACACGTCAT<br>CAACCGCTAGAGCGGACAAGAACTTCTTCAAGTGGACTATCAACACACGGTCCTTTGTGCAACTGCGT<br>AGGAAACTGGAGCTCTTCACGTACCTCAGATTTGACGCGGAGATTACCATTCTCACCACTGTGGCTGTT<br>AACGGCAGTGGAAACAATACTTATGTCGGTCTGCCTGACCTGACCTTGCAGGCAATGTTTGTACCGAC<br>AGGAGCCCTTACTCCCGAGAAACAGGACAGCTTTCATTGGCAGTCAGGCAGCAATGCTAGTGTGTTCT<br>TCAAGATCAGTGATCCACCAGCTCGGATTACCATCCCCTTCATGTGCATCAACTCTGCCTATAGCGTTT<br>TCTATGACGGCTTTGCCGGATTTGAGAAGAATGGGCTGTATGGGATTAACCCTGCTGACACAATTGGC<br>AATCTGTGTGTGCGGATCGTGAATGAGCACCAACCAGTGGGGTTTACAGTGACGGTGCGAGTGTACAT<br>GAAGCCCAAACACATCAAGGCATGGGCACCCAGACCTCCTAGGACTCTTCCGTACATGAGCATAGCCA<br>ACGCGAACTACAAAGGGAAAGAGCGAGCCCCAAATGCACTGTCTGCCATAATCGGTAACAGGGATTCT<br>GTCAAGACCATGCCCCACAATATCGTCAACACCTGA
EV-D68 MO VP1<br>with human IgG κchain<br>secretory signal peptide	ATGGAAACCGATACCCTCCTCTTGTGGGTCCTTCTGCTTTGGGTGCCTGGGAGCACTGGCGATCTGGA<br>TCACCTTCATGCAGCAGAAGCTGCCTACCAGATTGAGTCCATCATCAAGACAGCCACAGATACCGTGA<br>AATCCGAGATAAACGCTGAACTGGGTGTTGTTCCATCCCTGAATGCAGTAGAAACAGGAGCCACTTCC<br>AATACCGAACCTGAAGAGGCCATTCAGACACGCACCGTGATTAACCAGCATGGCGTAAGCGAAACTCT<br>GGTCGAGAATTTCCTCTCTCGCGCTGCCTTGGTCAGCAAACGCAGCTTCGAATACAAGGATCACACGT<br>CATCAACCGCTAGAGCGGACAAGAACTTCTTCAAGTGGACTATCAACACACGGTCCTTTGTGCAACTG<br>CGTAGGAAACTGGAGCTCTTCACGTACCTCAGATTTGACGCGGAGATTACCATTCTCACCACTGTGGC<br>TGTTAACGGCAGTGGAAACAATACTTATGTCGGTCTGCCTGACCTGACCTTGCAGGCAATGTTTGTACC<br>GACAGGAGCCCTTACTCCCGAGAAACAGGACAGCTTTCATTGGCAGTCAGGCAGCAATGCTAGTGTGT<br>TCTTCAAGATCAGTGATCCACCAGCTCGGATTACCATCCCCTTCATGTGCATCAACTCTGCCTATAGCG<br>TTTTCTATGACGGCTTTGCCGGATTTGAGAAGAATGGGCTGTATGGGATTAACCCTGCTGACACAATTG<br>GCAATCTGTGTGTGCGGATCGTGAATGAGCACCAACCAGTGGGGTTTACAGTGACGGTGCGAGTGTA<br>CATGAAGCCCAAACACATCAAGGCATGGGCACCCAGACCTCCTAGGACTCTTCCGTACATGAGCATAG<br>CCAACGCGAACTACAAAGGGAAAGAGCGAGCCCCAAATGCACTGTCTGCCATAATCGGTAACAGGGA<br>TTCTGTCAAGACCATGCCCCACAATATCGTCAACACCTGA

Table S1. *Continued*

Gene	Sequence
EV-D68 P1	ATGGGCGCACAAGTAACCCGCCAGCAGACTGGTACTCACGAGAACGCAAACATCGCCACTAATGGGT<br>CACACATCACCTATAACCAGATAAATTTCTACAAAGACTCCTATGCTGCATCCGCATCCAAACAAGATTT<br>CTCACAGGATCCCTCTAAGTTCACTGAACCCGTGGTGGAGGGCCTGAAAGCCGGAGCACCAGTGCTT<br>AAGTCCCCATCTGCCGAGGCGTGTGGCTACTCCGACCGAGTATTGCAGCTGAAACTTGGGAATAGTGC<br>CATCGTGACTCAGGAAGCTGCGAATTACTGCTGCGCCTATGGCGAATGGCCCAATTATCTGCCAGATC<br>ATGAGGCAGTCGCTATTGACAAGCCAACACAACCAGAAACCGCAACAGACAGGTTTTACACGCTGAAA<br>TCCGTGAAGTGGGAAACCGGTAGCACAGGATGGTGGTGGAAACTGCCTGATGCACTCAACAACATTG<br>GGATGTTCGGGCAGAATGTGCAGCACCATTACCTGTACAGAAGCGGGTTTCTGATACACGTGCAGTGC<br>AATGCTACAAAGTTTCACCAAGGCGCTCTCCTGGTTGTAGCCATTCCCGAGCATCAGAGAGGAGCACA<br>CAATACTAATACTAGCCCTGGGTTCGACGATATCATGAAAGGGGAGGAAGGAGGCACATTCAATCATC<br>CCTATGTCTTGGATGACGGCACGTCACTTGCGTGTGCTACTATATTTCCACACCAGTGGATAAATCTGC<br>GCACAAACAACAGCGCTACTATTGTGCTGCCCTGGATGAACGCTGCACCCATGGACTTCCCACTGCGG<br>CATAATCAGTGGACTCTGGCCATAATCCCTGTAGTGCCTCTTGGAACTCGTACCACGTCTAGCATGGTT<br>CCCATCACGGTGTCTATTGCGCCGATGTGTTGCGAATTCAATGGCCTGCGACATGCGATTACACAGGG<br>AGTCCCAACCTACCTCCTCCCAGGGAGTGGACAGTTTCTCACTACCGATGACCACAGCAGTGCCCCAG<br>CTCTGCCTTGCTTCAACCCAACTCCGGAGATGCACATTCCTGGGCAAGTGCGCAATATGCTGGAGGTG<br>GTTCAGGTCGAGTCTATGATGGAGATCAACAATACCGAGTCTGCCGTGGGTATGGAGCGACTCAAAGT<br>GGACATCTCCGCTCTGACAGACGTTGACCAACTGCTGTTTAACATTCCCCTCGACATCCAGCTCGATG<br>GACCCTTGCGCAACACCTTGGTGGGGAACATAAGTCGCTACTACACCCATTGGAGCGGGAGCCTGGA<br>AATGACCTTTATGTTCTGTGGGAGCTTTATGGCTGCAGGCAAGCTGATACTGTGCTATACCCCTCCTGG<br>CGGATCATGTCCCACAACCAGGGAGACAGCCATGCTGGGAACCCACATTGTGTGGGACTTTGGTCTG<br>CAATCCTCCGTGACTCTCATCATCCCTTGGATTAGTGGCTCACACTACCGGATGTTTAACAACGATGCC<br>AAATCCACCAACGCTAATGTGGGATATGTGACTTGTTTCATGCAGACTAACCTCATTGTGCCCTCTGAG<br>AGCAGCGATACTTGCTCACTTATCGGCTTCATTGCGGCCAAGGATGACTTTTCTCTTAGGCTGATGCG<br>GGACTCTCCGGATATCGGACAACTGGACCATCTGCATGCTGCCGAGGCCGCTTACCAGATTGAAAGCA<br>TAATCAAGACAGCGACAGACACAGTAAAGTCCGAGATTAACGCAGAGCTGGGTGTTGTGCCCTCACTC<br>AACGCTGTCGAAACAGGAGCCACCTCCAACACAGAACCTGAAGAAGCCATCCAGACCAGAACCGTTAT<br>CAACCAGCATGGCGTGAGCGAGACATTGGTCGAAAACTTTCTGTCCAGGGCCGCATTGGTCAGCAAG<br>CGTAGCTTTGAGTACAAGGATCACACGAGCAGCACAGCTAGAGCCGATAAGAACTTCTTTAAGTGGAC<br>GATTAATACCCGGAGTTTCGTTCAGCTGCGCCGGAAACTGGAACTGTTTACATATCTCAGGTTTGATGC<br>CGAGATTACCATTCTGACCACGGTCGCCGTCAATGGTTCTGGTAATAACACCTATGTGGGGCTTCCTG<br>ACCTGACCTTGCAGGCTATGTTTGTGCCTACTGGCGCACTGACCCCGGAAAAGCAGGACAGTTTCCAT<br>TGGCAGAGTGGCTCTAACGCCAGCGTGTTCTTCAAGATCTCCGACCCTCCCGCTAGAATCACGATCCC<br>ATTCATGTGCATCAACTCAGCCTATTCAGTATTCTATGATGGCTTTGCCGGTTTCGAGAAGAATGGCCT<br>GTACGGAATCAATCCAGCAGATACAATCGGCAATCTCTGTGTCCGTATTGTCAATGAGCACCAACCCG<br>TCGGTTTCACCGTTACAGTGAGGGTTTACATGAAGCCGAAACACATCAAAGCCTGGGCACCCAGACCT<br>CCGCGGACCCTTCCTTATATGTCTATCGCCAACGCTAACTACAAAGGCAAAGAGAGAGCCCCAAATGC<br>CTTGAGTGCCATCATTGGGAATAGGGATAGCGTTAAGACGATGCCCCACAACATAGTCAATACATGA
EV-D68 3CD	ATGGGCCCTGGGTTTGATTTCGCACAAGCCATCATGAAGAAGAACACAGTCATCGCTCGGACAGAGAA<br>GGGGGAGTTCACTATGCTGGGCGTATACGATAGAGTTGCGGTGATTCCCACTCATGCCAGTGTGGGG<br>GAAACGATCTACATCAACGATGTCGAAACAAAGGTGCTGGACGCATGTGCTCTGAGAGACTTAACGGA<br>CACTAACCTGGAGATCACCATTGTGAAGCTGGATCGGAACCAGAAATTTAGAGACATTCGCCATTTCCT<br>GCCCAGGTACGAAGATGACTATAATGACGCTGTATTATCTGTACACACGAGTAAATTCCCGAACATGTA<br>CATTCCCGTTGGCCAAGTCACCAATTACGGGTTTCTTAACCTTGGTGGCACTCCTACCCATAGGATCCT<br>CATGTACAATTTCCCTACACGAGCCGGACAGTGTGGTGGTGTAGTGACAACAACAGGGAAGGTGATTG<br>GGATACACGTCGGTGGTAATGGCGCTCAAGGATTCGCCGCAATGCTCCTTCACTCCTACTTCAGTGAT<br>ACTCAGGGAGAGATCGTTAGCAGCGAAAAGAGCGGAGTGTGCATTAACGCTCCAGCGAAGACCAAAC<br>TCCAGCCTTCTGTGTTTCATCAGGTGTTTGAGGGAAGTAAAGAACCAGCGGTACTAAACCCCAAAGATC<br>CACGCCTCAAGACCGACTTTGAGGAGGCCATTTTCTCCAAATACACGGGAAATAAGATCATGCTGATG<br>GACGAGTACATGGAAGAAGCTGTGGACCACTATGTGGGGTGTCTGGAACCACTCGATATCTCCGTGGA<br>TCCGATCCCACTTGAGTCAGCCATGTACGGTATGGATGGACTTGAAGCACTTGACTTGACCACCTCTG<br>CAGGCTTTCCCTACCTGCTACAGGGGAAGAAAAAAAGGGACATATTCAACCGACATACTAGGGACACC<br>TCTGAGATGACTAAAATGCTGGAGAAATATGGCGTTGATTTACCTTTCGTCACCTTTGTCAAAGATGAG<br>CTGCGATCTCGTGAGAAGGTGGAAAAAGGCAAGAGCCGTTTGATTGAAGCCTCGTCTCTGAACGACTC<br>TGTTGCCATGCGCGTTGCCTTTGGCAATTTGTATGCCACATTTCACAATAATCCTGGCACAGCTACTGG<br>GTCAGCCGTGGGATGTGACCCCGACATCTTCTGGTCCAAAATCCCGATCCTCCTGGATGGCGAGATTT<br>TCGCGTTTGACTATACCGGCTATGATGCTTCCTTGAGCCCTGTGTGGTTCGCATGCTTGAAGAAAGTCC<br>TGATTAAGCTGGGTTACACCCATCAGACTTCCTTTATCGACTACCTCTGCCACAGTGTGCACCTCTACA<br>AGGATAAGAAGTACATCGTGAATGGCGGTATGCCAAGTGGATCCTCAGGAACCTCAATCTTCAACACC<br>ATGATCAACAATATCATCATCCGCACACTGCTGATACGGGTTTATAAGGGCATTGACCTGGATCAGTTC<br>AAGATGATAGCCTATGGAGATGATGTGATAGCCTCGTATCCCCATAAGATAGATCCCGGGTTACTTGCT<br>GAGGCAGGAAAGCAGTATGGGCTCGTCATGACACCTGCCGACAAGGCACGAGCTTCATTGACACCA<br>ATTGGGAGAATGTGACTTTTCTAAAGCGGTACTTTCGCGCAGACGACCAATACCCCTTCCTAATTCACC<br>CAGTCATGCCAATGAAAGAGATTCACGAAAGCATTCGGTGGACCAAAGACCCCAGAAACACACAGGAT<br>CATGTCAGGAGCCTGTGCTATCTGGCATGGCACAATGGCGAAGAAGCCTATAACGAGTTCTGCCGCAA<br>AATACGGTCAGTGCCAGTTGGGAGAGCCTTGACTCTCCCTGCTTATTCCAGCCTGAGAAGGAAGTGGT<br>TGGACTCATTTTGA
