# Supplementary material for: mRNA vaccine expressing enterovirus D68 virus-like particles induces potent neutralizing antibodies and protects against infection
Source: Mol Ther Nucleic Acids. 2025 Oct 6;36(4):102731. doi: 10.1016/j.omtn.2025.102731 (PMC12593654; doi:10.1016/j.omtn.2025.102731)
Supplement: Document S2. Article plus supplemental information [file mmc2.pdf]

Molecular Therapy
Nucleic Acids
Original Article

mRNA vaccine expressing enterovirus D68 virus-like particles induces potent neutralizing antibodies and protects against infection

Yuta Kunishima,[1,2] Kota Senpuku,[1,3] Chikako Kataoka-Nakamura,[2] Toshiro Hirai,[1,3,4,5] and Yasuo Yoshioka[1,2,3,4,5,6,7]

[1]Vaccine Creation Group, BIKEN Innovative Vaccine Research Alliance Laboratories, Research Institute for Microbial Diseases, The University of Osaka, 1-6 Yamadaoka, Suita, Osaka 565-0871, Japan; [2]The Research Foundation for Microbial Diseases of Osaka University, 3-1 Yamadaoka, Suita, Osaka 565-0871, Japan; [3]Laboratory of Nano-Design for Innovative Drug Development, Graduate School of Pharmaceutical Sciences, The University of Osaka, 1-6 Yamadaoka, Suita, Osaka 565-0871, Japan; [4]Vaccine Creation Group, BIKEN Innovative Vaccine Research Alliance Laboratories, Institute for Open and Transdisciplinary Research Initiatives, The University of Osaka, 3-1 Yamadaoka, Suita, Osaka 565-0871, Japan; [5]Center for Advanced Modalities and DDS, The University of Osaka, 3-1 Yamadaoka, Suita, Osaka 565-0871, Japan; [6]Global Center for Medical Engineering and Informatics, The University of Osaka, 3-1 Yamadaoka, Suita, Osaka 565-0871, Japan; [7]Center for Infectious Disease Education and Research, The University of Osaka, 3-1 Yamadaoka, Suita, Osaka 565-0871, Japan

Enterovirus D68 (EV-D68) causes respiratory illness in children. It also causes severe paralysis called acute flaccid myelitis (AFM), which has become a global health threat. Here, we generated an mRNA vaccine expressing virus-like particles (VLPs) of EV-D68. We found that the mRNA vaccine elicited potent neutralizing antibodies against EV-D68 in the blood, and the neutralizing titer was superior to that of the inactivated whole virion (IWV) vaccine. The mRNA vaccine showed protective effects against intranasal challenge with EV-D68, and antisera from the vaccinated mice prevented the paralysis caused by EV-D68 infection in neonatal mice. Moreover, the mRNA vaccine induced neutralizing antibodies in the respiratory tract, which is the entry site for EV-D68. Additionally, it attenuated infection with coxsackievirus B3 (CVB3), which belongs to another enterovirus group, via CD8$^+$ T cell responses. In conclusion, our results suggest that this mRNA vaccine is a promising candidate for EV-D68 prevention.

INTRODUCTION

Enterovirus D68 (EV-D68) is a non-polio enterovirus first isolated in the United States in 1962.[1] Since the global outbreak of over 2,000 cases in 2014, EV-D68 has continued to circulate worldwide.[2] Unlike many enteroviruses, which are transmitted via the fecal-oral route, EV-D68 is transmitted through the respiratory route and causes respiratory illnesses, mainly in children. Symptoms range from mild, such as coughing and wheezing, to severe, such as pneumonia and respiratory distress.[3] Moreover, EV-D68 is as an emerging cause of acute flaccid myelitis (AFM), which develops paralysis rapidly over a period of hours to days due to the development of gray matter lesions.[4] The incidence ratio of AFM by EV-D68 remains unclear because the number of EV-D68 patients is not accurately estimated, but EV-D68 has been reported to be the most common enterovirus detected in AFM-affected patient specimens; the number of EV-D68-positive samples in the United States was 24 of 143 AFM samples tested in 2016 and 29 of 223 in 2018.[5] Therefore, EV-D68 is considered a health-threatening disease globally, and the development of an effective vaccine is required.

EV-D68 is a non-enveloped virus, and its capsid consists of 60 protomers assembled from four structural proteins, VP1–VP4. The viral protease 3CD cleaves the capsid precursor P1, dividing it into the capsid proteins VP0, VP1, and VP3.[6,7] Subsequently, the process of encapsulating the viral RNA to form a mature virion is linked to the cleavage of VP0 into VP2 and VP4. Of these structural proteins, VP1, VP2, and VP3 are exposed on the surface layer of the capsid, whereas VP4 is located inside the capsid. Although the host cell receptor is not fully identified, it is believed that sialic acid on the cell surface binds through the canyon region spanning VP1/2/3 and that this region is critical for cell infection.[8] Moreover, heparan sulfate glycosaminoglycans and intercellular adhesion molecule 5 on the cell surface are believed to facilitate the viral entry in certain circumstances.[9,10] Recently, major facilitator superfamily domain containing protein 6 (MFSD6) has been reported as an entry receptor of EV-D68, as MFSD6 was found to interact with the canyon region of EV-D68 at a distinct site of sialic acid-binding region.[11,12] The neutralizing epitopes of the virus are distributed on VP1/2/3. In particular, several linear or conformational epitopes are present in VP1, such as the BC loop, DE loop, and C-terminal region, and VP1 has been reported as a vaccine target for other enteroviruses.[13–17]

Received 8 May 2025; accepted 2 October 2025;
https://doi.org/10.1016/j.omtn.2025.102731.

Correspondence: Yasuo Yoshioka, PhD, Vaccine Creation Group, BIKEN Innovative Vaccine Research Alliance Laboratories, Research Institute for Microbial Diseases, The University of Osaka, 1-6 Yamadaoka, Suita, Osaka 565-0871, Japan.
E-mail: y-yoshioka@biken.osaka-u.ac.jp

© 2025 The Author(s).
Published by Elsevier Inc. on behalf of The American Society of Gene and Cell Therapy.
This is an open access article under the CC BY license (http://creativecommons.org/licenses/by/4.0/).

Currently, there are no specific anti-viral treatments or vaccines available against EV-D68. The enterovirus vaccines that are in current clinical use are live vaccines against polioviruses and the inactivated whole virion (IWV) vaccine against enterovirus A71 (EV-A71).[18] The live polio vaccine is an oral vaccine that uses the non-pathogenic Sabin strain.[19] However, vaccine strain-derived poliomyelitis is considered a problem due to genetic reversion.[20] In contrast, IWV vaccines for poliovirus and EV-A71 have shown efficacy and safety in clinical use; however, the productivity of these vaccines varies significantly depending on the virus strain, which can be a barrier to the rapid development of vaccines against emerging epidemic strains.[21,22] Virus-like particles (VLPs) are also considered as effective vaccine modalities that do not use viruses. Enterovirus VLPs are capsid-mimicking particles that do not contain the viral genome. When the capsid precursor P1 and its processor 3CD are expressed in the same cell, P1 is cleaved by 3CD to form a VLP, although genome inclusion and cleavage of VP0 into VP2 and VP4 does not occur. Recombinant VLP vaccines have shown efficacy against EV-D68, EV-A71, and coxsackievirus A6 (CV-A6) in mice.[23–25]

Recently, mRNA vaccines have emerged as a potent vaccine modality. mRNA vaccines are administered as mRNA-lipid nanoparticles (LNPs), in which the mRNA encoding the antigen is encapsulated within LNPs that serve as carriers and adjuvants.[26] mRNA vaccines induce antibody and CD8$^+$ T cells strongly, as demonstrated by their contribution to suppressing the SARS-CoV-2 pandemic.[27] In vaccine development, a significant benefit of the mRNA vaccine is its capacity to apply any antigen without culturing target pathogens. In addition to SARS-CoV-2, mRNA vaccines against the respiratory syncytial virus (RSV) have been approved by the US Food and Drug Administration,[28] and mRNA vaccines are being developed against a variety of viruses, including influenza,[29] HIV,[30] cytomegalovirus,[31] and Zika virus.[32,33] However, it is still unclear whether mRNA vaccines can be used against enteroviruses.

In this study, we aimed to investigate the efficacy of an mRNA vaccine expressing EV-D68 VLPs in mice. Our findings provide crucial insights revealing the potential of an mRNA vaccine expressing VLP as an mRNA vaccine platform for EV-D68.

RESULTS

mRNA vaccine for VP1 did not induce neutralization antibodies against EV-D68

We first examined the potential of an mRNA vaccine against VP1. We used VP1 from the MO strain (US/MO/14-18947: Clade B1) as an antigen. There were no secretory signals for VP1, and the expressed VP1 antigen remained intracellular. Therefore, we used the mRNA encoding VP1 with a secretory signal peptide derived from human IgGκ or interleukin (IL)-2 for extracellular secretion (Figure 1A). Agarose gel electrophoresis revealed a sufficient purity of the synthesized mRNA (Figure S1A). To examine the expression of VP1 in cells, mRNAs were transfected into HEK293 cells, and the cell lysates and supernatants were analyzed by western blotting with an anti-VP1 primary antibody. In the case of VP1 without secretory signal peptides, we detected a weak band at the same position as the virus-derived VP1 in the cell lysate, but not in the culture supernatant (Figures 1B and S2A). However, for the VP1 with secretory signal peptides, we detected strong bands of VP1, especially VP1 with secretory signal peptide derived from IL-2, not only in the cell lysate but also in the culture supernatant, although the bands were approximately 10 kDa higher than those of the virus-derived VP1 (Figure 1B).

To evaluate the efficacy of the mRNA vaccine against VP1, the mRNAs were encapsulated in LNP and intramuscularly administered to mice twice (Figure 1C). The mRNA vaccine for VP1 without the secretory signal peptide did not induce significant levels of EV-D68-specific IgG (Figure 1D) or neutralizing antibodies (Figure 1E) against EV-D68 in the blood compared with the non-vaccine group. In contrast, the mRNA vaccines for VP1 with secretory signal peptides induced significantly higher EV-D68-specific IgG in the blood than the non-vaccine group but did not induce any neutralizing antibodies (Figures 1D and 1E). These results suggest that the VP1 mRNA is not a promising antigen for EV-D68.

mRNA vaccine expressing VLP induced neutralization antibodies against EV-D68

Next, we evaluated an mRNA vaccine expressing VLP (Figure 2A). We used mRNAs encoding the structural protein P1 and the non-structural protein 3CD of EV-D68 (Figure S1B). To examine whether VLP was expressed, these mRNAs were mixed at a mass ratio of 1:1 and transfected into HEK293 cells. Western blotting results of the cell lysate and supernatant showed that VP1 was expressed at almost the same molecular weight as those derived from the virus (Figures 2B and S2B), indicating that 3CD processed P1. To confirm the formation of VLPs, the culture supernatant was analyzed by sucrose density gradient centrifugation, which can separate particles according to their sedimentation velocity. As a control, unpurified EV-D68 virus was analyzed by density gradient centrifugation, and VP1 was detected as a double peak (Figures 2C and S2C). As previously reported in our studies,[34] the higher-density band was presumed to correspond to full particles, while the lower one was presumed to correspond to empty capsids with a corresponding density to VLPs. In the culture supernatant transfected with mRNA encoding P1 and 3CD, VP1 was detected in the fraction corresponding to empty viral particles, suggesting that VLP had formed in the cells after transfection of P1 and 3CD mRNA (Figures 2C and S2D). Next, to confirm the formation of VLP *in vivo*, we prepared P1 mRNA-LNP and 3CD mRNA-LNP, mixed at a ratio of 1:1, and the mix was injected intramuscularly into mice. Western blotting analysis showed a band corresponding to VP1 in the lysate of the injected muscle, indicating that P1 was processed by 3CD and VLP was expressed *in vivo* (Figures 2D and S2E).

To evaluate the immunogenicity of this mRNA vaccine, we immunized mice two times with the VLP-expressing mRNA vaccine (Figure 2E). The mRNA vaccine significantly induced the production of anti-EV-D68 IgG (Figure 2F) and neutralizing antibodies

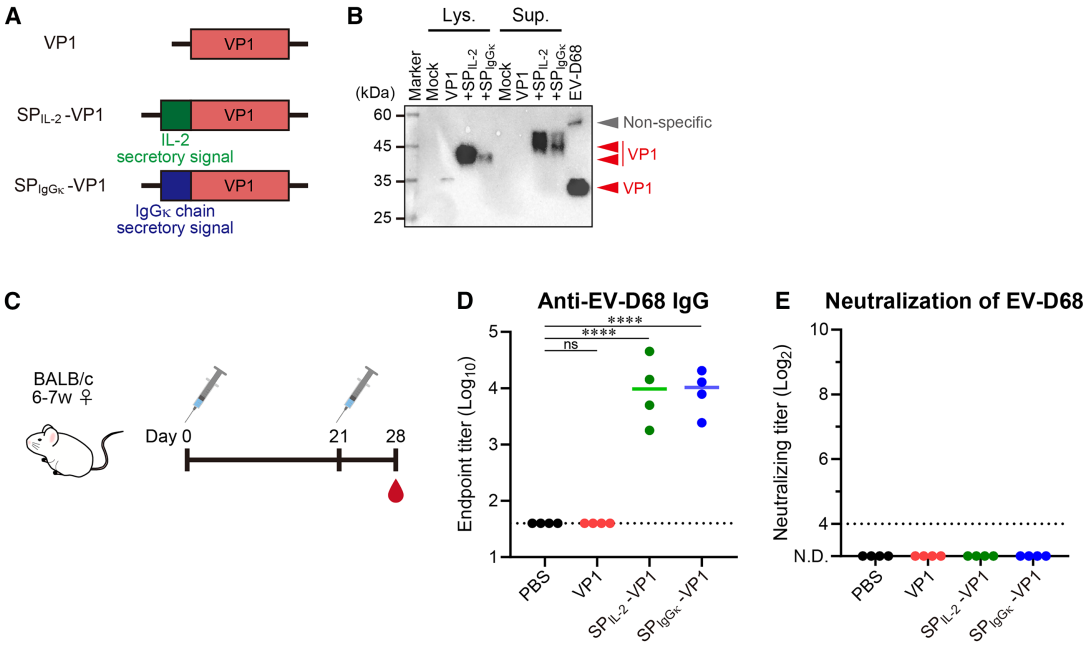

Figure 1. mRNA vaccine for VP1 induced anti-EV-D68 IgG but not neutralizing antibodies against EV-D68

(A) Schematic representation of the evaluated mRNAs. VP1 (red) is derived from a part of the structural protein of the EV-D68 MO strain. SP_{IL-2}-VP1 or $SP_{IgG\kappa}$-VP1 has secretory signal peptide derived from IL-2 (green) or IgGκ (blue) on the N terminus of VP1, respectively. (B) *In vitro* expression analysis of VP1. VP1 mRNA was transfected into HEK293 cells, and the culture supernatant or cell lysate was analyzed by western blotting with an anti-EV-D68 VP1 polyclonal antibody. The lane of EV-D68 means virus as a positive control. The viral VP1 is indicated by a red arrowhead, and the non-specific band is indicated by a gray arrowhead. (C) Vaccination and experiment schema of (D and E). Mice were vaccinated intramuscularly on days 0 and 21, and the plasma was collected on day 28. All the doses contained 2 μg mRNA. (D) Anti-EV-D68 IgG in plasma evaluated with ELISA. (E) Neutralizing antibody titers against EV-D68 measured on day 28. (D and E) Points are presented as individual data, and lines are presented as the median. Dotted line indicates the detection limit. $^{****}p < 0.0001$; N.D., not detectable; ns, not significant as indicated by Tukey's test.

against EV-D68 (Figure 2G). In contrast, vaccination with P1 or 3CD mRNA-LNPs did not induce the production of anti-EV-D68 IgG (Figure 2F) or neutralizing antibodies (Figure 2G). We then investigated the mixing ratio of P1 and 3CD mRNA-LNPs. The anti-EV-D68 IgG was highly induced at a P1:3CD mass ratio of 5:1 or 2:1 (Figure 2H). In contrast, neutralizing antibody titers against EV-D68 were not significantly different between 5:1 and 1:5, and the titers at 25:1 or 1:25 were lower than those between 5:1 and 1:5 (Figure 2I). These results indicate that VLP-expressing mRNA vaccines can induce EV-D68-specific IgG and neutralizing antibodies.

To investigate the protective effect of the mRNA vaccine against EV-D68, we challenged the mice by administering EV-D68 intranasally and quantified the viral titers in the nasal turbinate and lungs at 12 h post-challenge (Figure 3A). In the nasal turbinate and lungs of mice treated with the vaccine, the virus titer was significantly lower than that in non-vaccinated mice and was almost below the detection limit (Figure 3B). To evaluate whether the antibodies induced by the mRNA vaccine prevented paralysis, we used a passive serum transfer model in neonatal mice. The serum derived from the vaccinated mice was transferred to 1-day-old neonatal mice, and the mice were intraperitoneally challenged with EV-D68 the following day (Figure 3C). None of the mice treated with serum from vaccinated mice developed paralysis, while all mice treated with serum from non-vaccinated mice showed paralysis symptoms (Figure 3D). In addition, all mice transferred with vaccinated-mouse serum survived until day 14, whereas more than half of those transferred with non-vaccinated mouse serum died (Figure 3E). To evaluate whether the mRNA vaccine-induced antibodies prevent infection of the nervous system, we quantified the viral titers in the spinal cord at 4 days post-challenge (Figure 3C). In the spinal cord of mice treated with the vaccinated mouse serum, the virus titer was significantly lower than that in mice treated with non-vaccinated mouse serum (Figure 3F). These results indicate that the mRNA vaccine has the potential to prevent respiratory and neurological infection and neurological symptoms caused by EV-D68.

Non-neutralizing serum has been shown to elicit a protective effect in passive serum transfer models.[35] Hence, we confirmed whether the serum derived from mice vaccinated with VP1 with secretory signal peptides (SP_{IL-2}-VP1 or $SP_{IgG\kappa}$-VP1), P1, or 3CD, which failed to induce neutralizing antibodies (Figures 1E and 2G), showed protective effect for paralysis. Most of the mice transferred with these sera developed paralysis symptoms (Figure S3A), and more than half of those died until day 14 (Figure S3B). These results suggest that the vaccines, which failed to induce neutralizing antibodies, do not have the potential to prevent EV-D68-induced paralysis.

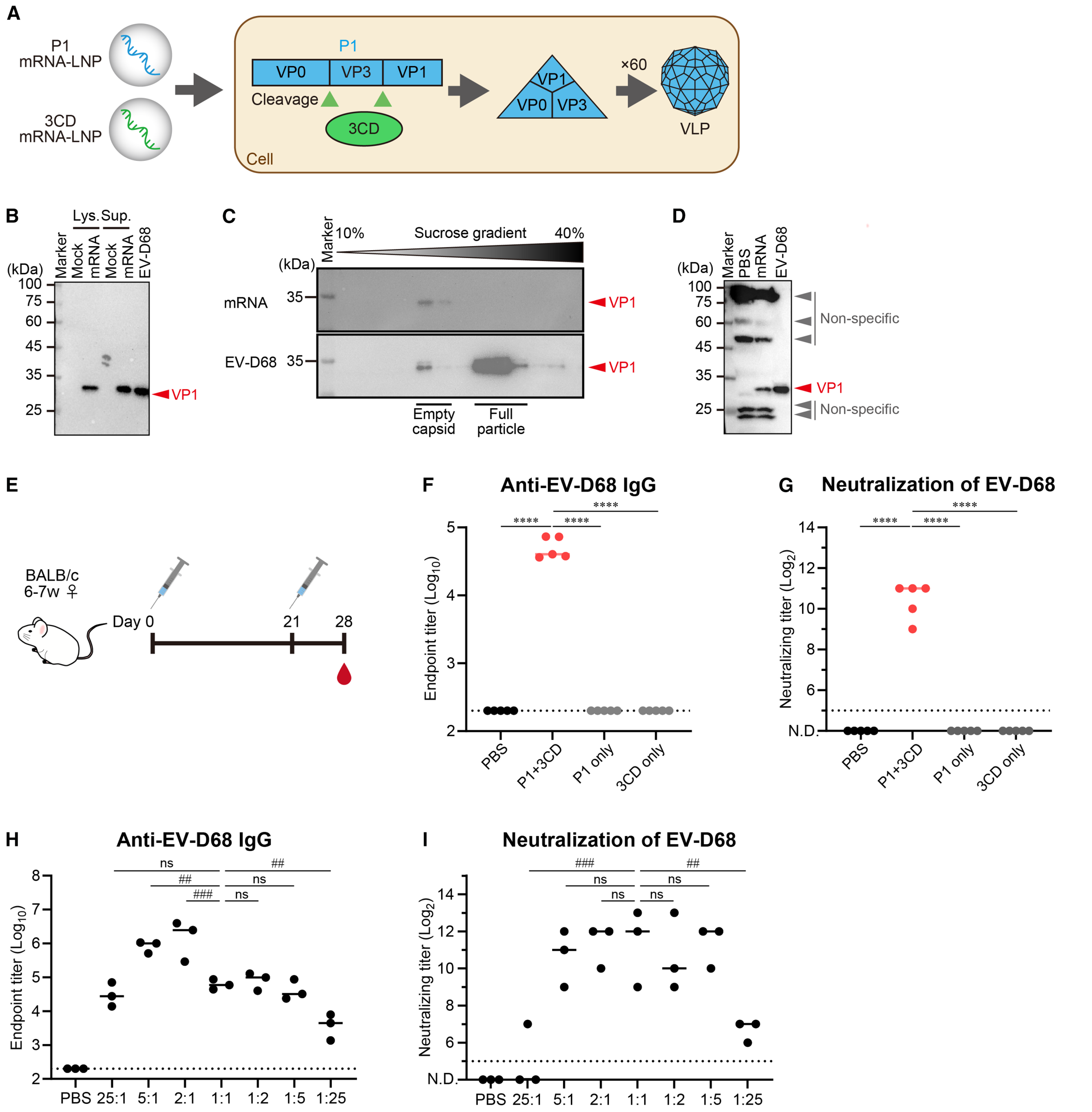

Figure 2. mRNA vaccine expressing VLP induced anti-EV-D68 IgG and neutralizing antibodies in blood

(A) Schematic representation of the mRNA vaccine expressing VLP. By co-administration of P1 mRNA-LNP and 3CD mRNA-LNP, P1 and 3CD were co-expressed in the cell. P1 was cleaved into VP0/VP3/VP1 by 3CD, and the VP0/VP3/VP1 were assembled into VLPs. (B) *In vitro* expression analysis of EV-D68 VLP. The culture supernatants and cell lysates from HEK293 transfected with P1 and 3CD mRNA (1:1 on weight) were analyzed by western blotting using anti-EV-D68 VP1 polyclonal antibody. (C) Confirmation of VLP formation by density gradient. The culture supernatants from HEK293 transfected with P1 and 3CD mRNA (1:1 on weight) were subjected to 10%–40% sucrose gradient, following by western blotting using anti-EV-D68 VP1 polyclonal antibody. As control, culture supernatant of EV-D68-inoculated cells was tested in parallel. (D) *In vivo* expression analysis of EV-D68 VLP. Mixture of P1 mRNA-LNP and 3CD mRNA-LNP (1:1 on weight, total 2 μg of mRNA) was injected into muscle, and the expression of EV-D68 VLP was analyzed with western blotting using the anti-EV-D68 VP1 polyclonal antibody. (B and D) The lane of EV-D68 means virus as a positive control. (B–D) The viral VP1 is indicated by the red arrowheads, and the non-specific bands are indicated by the gray arrowheads. (E) Vaccination and experiment schema of (F–I). Mice were

(legend continued on next page)

mRNA vaccine expressing VLP induced higher levels of neutralization antibodies than IWV

To determine the potency of the mRNA vaccine expressing VLP in inducing antibodies, we compared the levels of anti-EV-D68 IgG and neutralizing antibody titers between the mRNA and IWV vaccines. IWV was prepared from EV-D68 viruses inactivated with β-propiolactone. We previously reported that IWV can protect against infection by EV-D68.[34] mRNA vaccines with 0.0032–2 μg of mRNA or IWV with 0.016–10 μg of protein were intramuscularly administered twice into mice. The mRNA vaccine induced significantly higher IgG antibody titers at doses of 2 μg than IWV at all doses examined, although the titer induced by the mRNA vaccine was reduced when a dose of 0.08 μg was used, and no IgG antibodies were detected below the administration of a 0.0016 μg dose (Figure 4A). Anti-EV-D68 IgG1, IgG2a, and IgG2b titers also showed similar trends, although the IgG1 and IgG2b titers induced by 2 μg of the mRNA vaccine were not significantly higher than those induced by the IWV (Figures 4B–4D). Next, the neutralizing antibody titer in the blood was examined. Consequently, 2 μg doses of the mRNA vaccine produced the highest neutralizing antibody titer (approximately 1,024) among the samples, while the neutralizing antibody titer induced by IWV was approximately 256, even when the dose was increased to 10 μg (Figure 4E). These results suggest that the mRNA vaccine can induce IgG and neutralizing antibodies to EV-D68 more effectively than the IWV vaccine.

To evaluate the persistence of antibodies induced by this vaccine, long-term trends in anti-EV-D68 IgG antibody titers after 2 μg of the mRNA vaccine were compared with those after 2 μg of IWV. The IgG antibody titers induced by the mRNA vaccine and IWV remained almost unchanged from the second dose until the end of the observation period, 5 months after the start of the experiment (Figure 4F). These results highlight that the durability of the antibody responses induced by the mRNA vaccine is comparable to that obtained using IWV.

To evaluate the cross-reactivity of antibodies induced by this vaccine against other strains, IgG antibody titers for US/IL/14-18952 (IL strain: clade B2) and US/KY/14-18953 (KY strain: clade D) in the blood were quantified. Anti-IL strain IgG titers were observed when 2 μg or 0.4 μg of mRNA vaccine was immunized, and the titer tended to be higher than that in the IWV-vaccinated groups (Figure 4G). Anti-KY strain IgG titers in the 2 μg or 0.4 μg mRNA vaccine groups were comparable to that of the maximum dose (10 μg) of IWV (Figure 4H). Cross-neutralizing antibodies against the IL strain tended to be at higher levels in the 2 μg of mRNA vaccine group than at any dose in the IWV vaccine group (Figure 4I). Neutralizing activity against the KY strain was observed in some mice at doses of 2 or 0.4 μg in the mRNA vaccine group, whereas neutralizing antibodies against the KY strain were not detected in all mice vaccinated with IWV (Figure 4J). Additionally, we evaluated cross-neutralizing antibodies against the recently isolated 1520-Yamagata-2015[36] and EVD68/Yamagata.JPN/2023-89[37] strains (clade B3). The levels of cross-neutralizing antibodies against both strains were significantly higher in the 2 μg of mRNA vaccine group than at any dose in the IWV vaccine group (Figures 4K and 4L). Finally, neutralizing antibody titers were compared after the third dose of the 2 μg of mRNA vaccine or 2 μg of IWV. Consistent with the results obtained after the second dose, neutralizing antibody titers against the MO strain were significantly higher in the mRNA vaccine group than in the IWV vaccine group (Figure 4M). Furthermore, the third dose significantly induced neutralizing antibodies against the KY strain in all the mice in the mRNA vaccine group, whereas only one mouse in the IWV vaccine group showed cross-neutralizing antibodies (Figure 4N). These results indicate that mRNA vaccines tend to be more cross-reactive than IWV vaccines against non-vaccine strains.

Mucosal IgG and IgA were induced by the mRNA vaccine expressing VLP

As EV-D68 is a pathogen transmitted through the respiratory tract, vaccine-inducing antibodies in the airway mucosa are useful for protection against infection. We investigated whether an mRNA vaccine expressing VLP could induce antibodies in the respiratory tract. After intramuscular vaccination with the mRNA or IWV vaccine, nasal washes and bronchoalveolar lavage fluid (BALF) were collected. During the nasal wash, the mRNA vaccine significantly induced anti-EV-D68 IgG, and the IgG titer was significantly higher than that induced by the IWV (Figure 5A). In addition, the mRNA vaccine significantly induced IgA in the nasal wash, whereas IWV did not induce nasal IgA, implying that this effect was unique to the mRNA vaccine (Figure 5A). To clarify whether antibody induction in the nasal wash was caused by EV-D68 VLP antigen or the LNP composition used, we immunized mice two times with SARS-CoV-2 spike mRNA in the same LNP. As a result, the anti-spike IgA as well as IgG was induced in the nasal wash, suggesting that these nasal responses could be attributed to mRNA-LNP composition, rather than the EV-D68 VLP antigen (Figure S4). In BALF, both mRNA and IWV vaccines induced anti-EV-D68 IgG, and the titer induced by the mRNA vaccine was significantly higher than that induced by IWV (Figure 5B). To determine whether mucosal antibodies can neutralize EV-D68, neutralizing antibody titers in BALF and nasal washes were assessed. The mRNA vaccine showed significantly higher neutralizing antibodies than the IWV in the nasal wash

vaccinated intramuscularly on days 0 and 21, and the plasma was collected on day 28. All the doses contain 2 μg mRNA. (F) Anti-EV-D68 IgG in plasma. P1 + 3CD indicates that the mixture of P1 mRNA-LNP and 3CD mRNA-LNP (1:1 on weight) was vaccinated, and P1 or 3CD only indicates only P1 mRNA-LNP or 3CD mRNA-LNP vaccination. (G) Neutralizing antibody titers against EV-D68 in plasma. (H, I). The immunogenicity of mRNA vaccines expressing VLP with different ratios of P1 and 3CD. The ratio of P1 mRNA-LNP and 3CD mRNA-LNP varied from 25:1 to 1:25 to ensure that the total dose of mRNA was 2 μg. (H) Anti-EV-D68 MO IgG in plasma. (I) Neutralizing antibody titers against EV-D68 in plasma. (F–I) Points are presented as individual data, and lines are presented as the median. Dotted lines indicate detection limits. (G and I) N.D., not detectable. (F, G) ****$p < 0.0001$; ns, not significant as indicated by Tukey's test. (H, I) ##$p < 0.01$; ###$p < 0.001$; ns, not significant as indicated by Dunnett's test against 1:1.

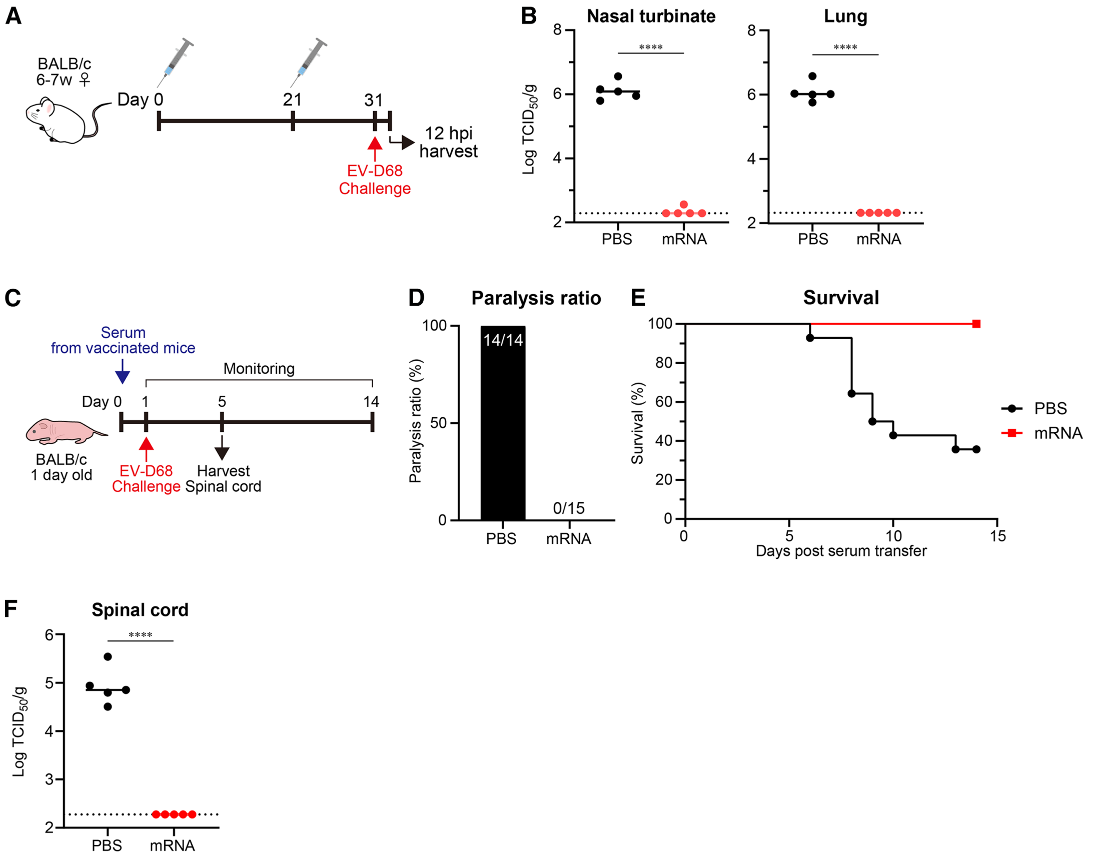

Figure 3. mRNA vaccine expressing VLP had the potential to protect from EV-D68 infection and paralysis

(A) Vaccination and experiment schema of (B). (B) The protection effect of the mRNA vaccine expressing VLP (P1:3CD = 1:1 on weight, total 2 μg of mRNA). Mice were intranasally challenged with the MO strain after vaccination, and viral titers in the nasal turbinate and lungs were determined. (C) Experiment schema to evaluate protective effect against infection in spinal cord and EV-D68-related paralysis. (D and E) The protecting effect of the antisera from vaccinated mice. Limb paralysis (D) and rate of survival (E) in the challenged mice were monitored every day for 14 days after the serum transfer. $n = 14$ in the control serum group, and $n = 15$ in the vaccinated serum group. (F) The viral titers in the spinal cord were evaluated 4 days after challenge. (B and F) Dotted lines indicate detection limits. ****$p < 0.0001$ as indicated by Student's t test.

(Figure 5C) and BALF (Figure 5D). These results indicate the high potential of this mRNA vaccine to induce IgA and IgG in the airway mucosa, even though the mucosal surface is not directly immunized.

To explore the source of nasal antibodies, we quantified the anti-EV-D68 antibody-secreting cells (ASCs) in the nasal cavity. Immune cells in the nasal tissue from vaccinated mice were collected and subjected to an enzyme-linked immunospot (ELISpot) assay. The results showed that IgG- and IgA-ASCs were detected in the nasal cavity with the mRNA vaccine, although the IgG-ASC count was not statistically significant compared with that in the PBS group, and IgG-ASCs and IgA-ASCs were not detected in one of the four mice (Figures 5E and 5F). In contrast, IgG- and IgA-ASCs were not significantly induced by IWV (Figures 5E and 5F). These results suggest that the nasal antibodies induced by the mRNA vaccine are partially derived from ASC induced in the nasal tissue. To investigate whether nasal antigen expression is involved in the induction of nasal antibodies, we quantified the expression and distribution of the antigen using mRNA-LNPs encoding firefly luciferase as a model. In addition to intramuscular injection, we administered mRNA-LNP intranasally as a control for nasal expression. After intramuscular administration, luciferase activity was detected in the nasal turbinate at statistically significant levels (Figures 5G and S5). Luciferase activity in the nasal turbinate was significantly higher after intranasal administration than after intramuscular injection (Figures 5G and S5). To confirm that nasal antigen expression resulted in the induction of nasal IgG and IgA, we intranasally administered VLP-expressing mRNA vaccines. Intranasal vaccination with mRNA vaccines did not induce nasal, blood, or BALF antibodies (Figure S6). These results suggest that factors other than antigen expression in nasal tissue are strongly involved in the induction of nasal antibodies by VLP-expressing mRNA vaccines.

$CD8^+$ T cell-mediated cross-protection against coxsackievirus B3 (CVB3)

Non-structural proteins, including 3CD, are more sequence-conserved within the enterovirus genus than structural proteins,

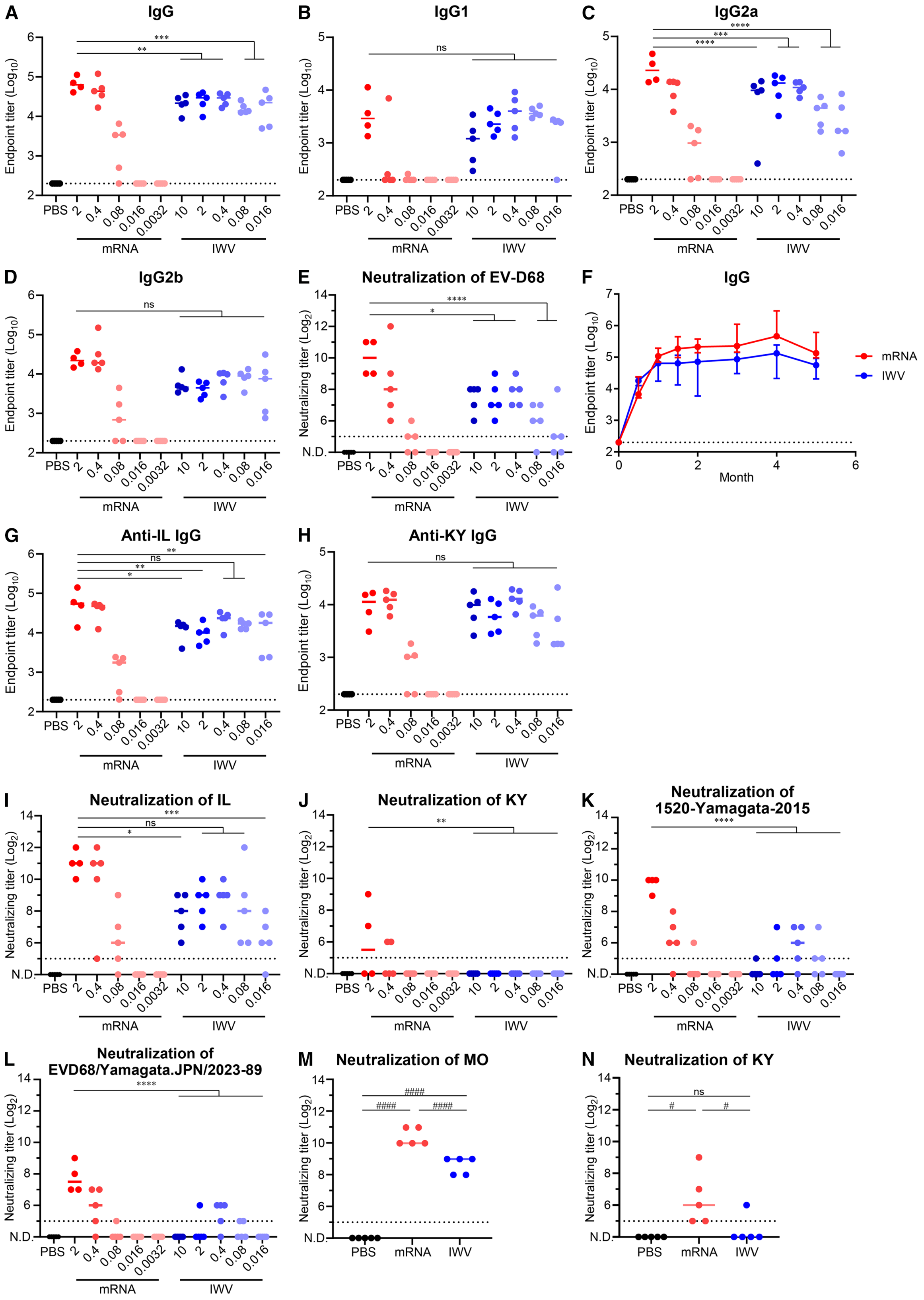

(legend on next page)

and the induction of the T cell response to this region might confer cross-protection against different strains.[38,39] Thus, we investigated whether the mRNA vaccine could induce a T cell response against 3CD of EV-D68. Following vaccination with two doses of mRNA vaccine or IWV, splenocytes were collected and restimulated with 3CD. With restimulation by 3CD, the interferon (IFN)-γ was significantly secreted in the mRNA vaccine group than in the IWV vaccine group (Figure 6A). In the mRNA vaccine group, IFN-γ was not significantly induced upon restimulation with 3C protein and significantly induced with 3D protein, indicating that induction of IFN-γ by 3CD is mainly attributed to the response to 3D (Figure 6A). These results indicate that the mRNA vaccine induced Th1 or $CD8^+$ T cell responses against the 3CD protein more potently than IWV.

Next, we investigated whether T cell responses to 3CD of EV-D68 could exert a protective effect against other enteroviruses. We used CVB3 as a mouse model for enterovirus challenge that differs from EV-D68. The 3CD mRNA-LNPs or P1 mRNA-LNPs for comparison were administered twice, and CVB3 was challenged intraperitoneally (Figure 6B). A significant decrease in body weight was observed in the non-vaccinated and P1 mRNA-LNP-vaccinated mice upon CVB3 challenge (Figure 6C). In contrast, weight loss was significantly suppressed in mice vaccinated with 3CD mRNA-LNPs compared with those vaccinated with PBS and P1 mRNA-LNPs (Figure 6C). To ascertain whether the inhibition of weight loss was T cell dependent, mice vaccinated with 3CD mRNA-LNPs were treated with anti-CD4 or anti-CD8 antibodies to deplete $CD4^+$ or $CD8^+$ T cells, respectively, and then challenged with CVB3. The weight change was not significantly different between mice depleted of $CD4^+$ T cells and the isotype control (Figure 6D). In contrast, the mice depleted of $CD8^+$ T cells lost weight (Figure 6E). These findings indicate that the mRNA vaccine may have a broad-spectrum protective effect by inducing $CD8^+$ T cells against 3CD.

DISCUSSION

In this study, we generated an mRNA vaccine expressing EV-D68 VLP *in vivo*. To the best of our knowledge, there have only been a few reports on vaccines expressing enterovirus VLPs *in vivo*. Tsou et al. reported that an adenovirus vector vaccine loaded with P1 and 3CD of EV-A71 could express VLP *in vivo* and induce neutralizing antibodies and cellular immunity against EV-A71.[40] In addition, Warner et al. recently reported that a self-amplifying RNA vaccine expressing EV-D68 VLPs can induce neutralizing antibodies against EV-D68 in the blood and protect against EV-D68 infection.[41] The self-amplifying RNA vaccine reported by Warner et al. consisted of a single RNA strand encoding a replicon derived from the Venezuelan equine encephalitis virus, P1 of EV-D68, IRES of encephalomyocarditis virus, and 3CD of EV-D68, in contrast to our mRNA vaccine, which consists of two mRNAs—P1 and 3CD. The design of a single RNA encoding P1 and 3CD is advantageous because it can reliably deliver P1 and 3CD into the same cell and does not require quality control of the two types of mRNA-LNP. However, the ratio of P1 mRNA-LNPs to 3CD mRNA-LNPs could be modified in our mRNA vaccine. The optimal P1:3CD ratio for the *in vitro* expression of enterovirus VLPs has been reported to vary among viruses.[42,43] Optimization of this ratio according to the target enterovirus species or clade may be important to achieve high immunogenicity. Moreover, we compared the antigenicity of the mRNA vaccine with IWV and examined the potential of the mRNA vaccine in the induction of mucosal antibodies and cross-protective effects mediated by $CD8^+$ T cells. The findings provide new insights, which have not been reported previously.

The mRNA vaccine expressing VLP showed a higher potential for induction of anti-EV-D68 IgG and neutralizing antibodies against EV-D68 than IWV. In addition, the mRNA vaccine induced a higher neutralizing activity against viral strains other than the vaccine strain, including recently isolated strains, suggesting that the vaccine could prevent infection of broad-spectrum EV-D68. It should be noted that this mRNA vaccine can, in principle, be generated regardless of a specific vaccine strain of enterovirus D68. In cases where the vaccine based on the MO strain does not exhibit efficacy against certain strains, it is highly likely that using a different vaccine strain could confer protective effects. When developing the vaccine, it is important to choose a vaccine strain from among the strains that are prevalent at the time. The reasons for high IgG and neutralizing antibody titers induced by the mRNA vaccine are unclear. Previous studies that evaluated EV-D68 VLP vaccines reported that VLPs induced potent neutralizing antibodies against EV-D68, but the titer was lower than that induced by an equivalent dose of IWV.[24] In addition, in reports of adenovirus vector vaccines expressing EV-A71 VLP, the maximum neutralizing antibody titers did not exceed those of IWV vaccines.[40] These reports imply that the induction of high neutralizing antibody titers with mRNA vaccines expressing VLP may be due to the characteristics of the mRNA vaccines. mRNA vaccines have been shown to strongly induce germinal center responses in the draining lymph nodes at the vaccination site, promote B cell proliferation and maturation, and induce antibodies of superior

Figure 4. Anti-EV-D68 IgG and neutralizing antibodies were induced more potently by the mRNA vaccine expressing VLP than IWV
(A–D) EV-D68 specific (A) IgG, (B) IgG1, (C) IgG2a, and (D) IgG2b titer after boost vaccination of the mRNA vaccine and IWV. The mRNA vaccine (2, 0.4, 0.08, 0.016, or 0.0032 μg of mRNA) or IWV (10, 2, 0.4, 0.08, or 0.016 μg of protein) immunizations were done twice, and anti-EV-D68 IgG in the plasma was evaluated with ELISA. (E) Neutralizing antibody titers against the homologous MO strain of EV-D68 after boost immunization with mRNA vaccine or IWV. (F) The persistence of anti-EV-D68 MO IgG antibodies in plasma. Data are shown as means ± SD. (G, H) The IgG titers against the heterologous (G) IL strain and (H) KY strain. (I–L) Neutralizing antibody titers against the (I) IL, (J) KY, (K) 1520-Yamagata-2015, and (L) EVD68/Yamagata.JPN/2023-89 strains. (M, N) Booster effect of the third dose. The neutralizing antibody titer against (M) MO strain and (N) KY strain in the plasma was quantified after the third vaccination. (E and I–N) N.D., not detectable. (A–E and G–N) Points are presented as individual data, and lines are presented as the median. (A–N) Dotted lines indicate detection limits. (A–E and G–L) *$p < 0.05$; **$p < 0.01$; ***$p < 0.001$; ****$p < 0.0001$; ns, not significant as indicated by Dunnett's test against 2 μg of mRNA. (M, N) #$p < 0.05$; ####$p < 0.0001$; ns, not significant as indicated by Tukey's test.

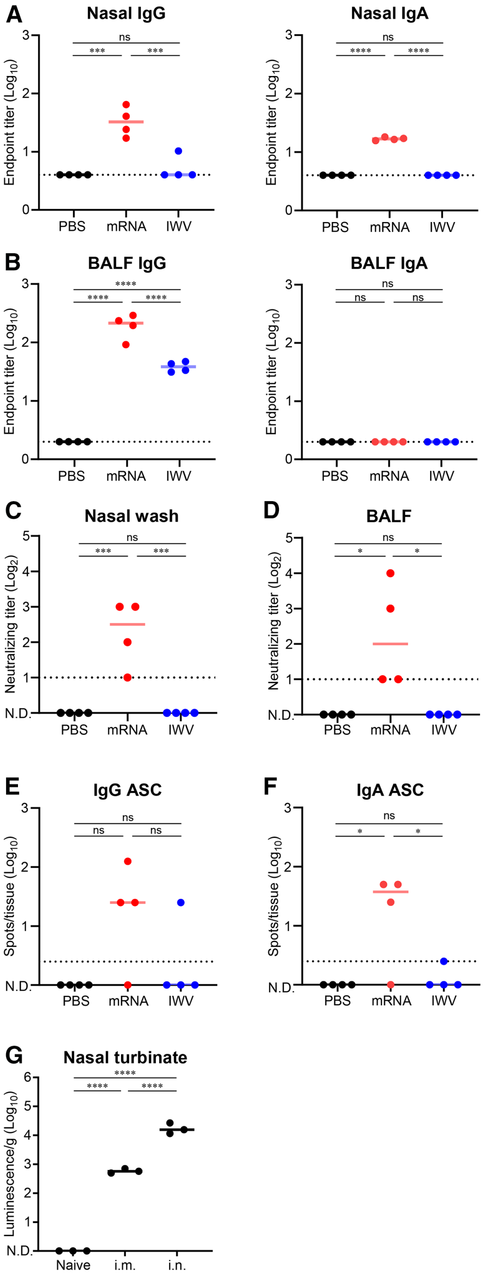

Figure 5. mRNA vaccine expressing VLP induced mucosal antibodies against EV-D68

(A–F) The mRNA vaccine (P1:3CD = 1:1 on weight, total 2 μg as mRNA) and IWV (2 μg as protein) were immunized twice, and the nasal wash, BALF, and immune cells in nasal tissue were collected. (A) Anti-EV-D68 IgG and IgA in the nasal wash. (B) Anti-EV-D68 IgG and IgA in the BALF. (C and D) Neutralizing antibody titers against EV-D68 in (C) nasal wash and (D) BALF. (E and F) The number of ASCs in the nasal tissue quantified by ELISpot assay. The number of (E) anti-EV-D68 IgG-ASC and (F) IgA-ASC were represented as spots per tissue. (G) The tissue distribution and expression of mRNA-LNP administrated intramuscularly or intranasally. The mRNA-LNP encoding firefly luciferase was administered to mice intramuscularly (i.m.) or intranasally (i.n.). The luciferase activity in the homogenate of nasal turbinate was quantified. The luminescence was standardized by tissue weight. (C–G) N.D., not detectable. (A–G) Points are presented as individual data, and lines are presented as the median. Dotted lines indicate detection limits. *$p < 0.05$; ***$p < 0.001$; ****$p < 0.0001$; ns, not significant as indicated by Tukey's test.

quantity and quality.[44–46] Further analysis of immune responses, such as the germinal center induced by this mRNA vaccine, will likely reveal the reason for the high neutralizing titer.

In general, the induction of antibodies on mucosal surfaces is thought to be beneficial for protection against infection, and mucosal vaccines have been developed to induce IgA antibodies on mucosal surfaces.[47] It has been directly demonstrated that secretory IgA in the nasal cavity contributes to protection against infection by certain pathogens, such as influenza[48,49] and RSV,[50] though the involvement of secretory IgA in protection against EV-D68 is not clear. Although parenteral vaccines are superior in systemically inducing IgG, there have been limited reports on the active induction of IgA antibodies on mucosal surfaces by these vaccines. For example, little IgA was detected in nasal washes after subcutaneous vaccination with EV-D68 IWV, SARS-CoV-2 IWV, or a recombinant spike protein vaccine.[34,51,52] In addition, in humans who received IWV injection of poliovirus, intestinal anti-poliovirus IgA was significantly lower than that in those who received the live oral vaccine.[53,54] In this study, we showed that intramuscular vaccination with our mRNA vaccine expressing VLP potently induced anti-EV-D68 IgA in the nasal wash, as well as anti-EV-D68 IgG in the nasal wash and BALF. With the parenteral IWV vaccine, IgA was not detected in the nasal wash, and IgG in the nasal wash and BALF was lower than that observed with the mRNA vaccines. In addition, the mRNA vaccine significantly induced mucosal neutralizing antibodies compared with the IWV vaccine. These results suggest that the mRNA vaccine may offer advantages over other parenteral vaccines in preventing EV-D68 mucosal infections. According to previous reports, IgG detected in BALF and nasal washes is transported from the blood to the alveoli via neonatal Fc receptors expressed on mucosal epithelial cells.[55,56] In addition, based on our ELISpot results, some of the IgG detected in the nasal wash may be from IgG-producing cells induced in the nasal cavity. However, the specific mechanism through which IgA is induced by intramuscular vaccines remains unclear. Based on the result of SARS-CoV-2 spike mRNA experiments, the mucosal IgA responses were suggested to be induced by mRNA-LNP composition, rather than EV-D68 VLP antigen. In humans, some reports have shown that intramuscular vaccination with SARS-CoV-2 mRNA induces spike-specific IgA in the nasal cavity and saliva,[57,58] though this remains controversial.[59,60] This implies that nasal IgA may have been induced by a mechanism specific to mRNA vaccines. Based on our luciferase mRNA experiments, it is possible that antigens enter the lymphoid tissue around the nose and induce IgA antibodies in the nasal mucosa. In contrast, nasal antigen expression was higher with intranasal administration than with intramuscular mRNA administration; however, intranasal immunization with the mRNA vaccine did not induce systemic or mucosal antibodies. This suggests that antigen expression in the

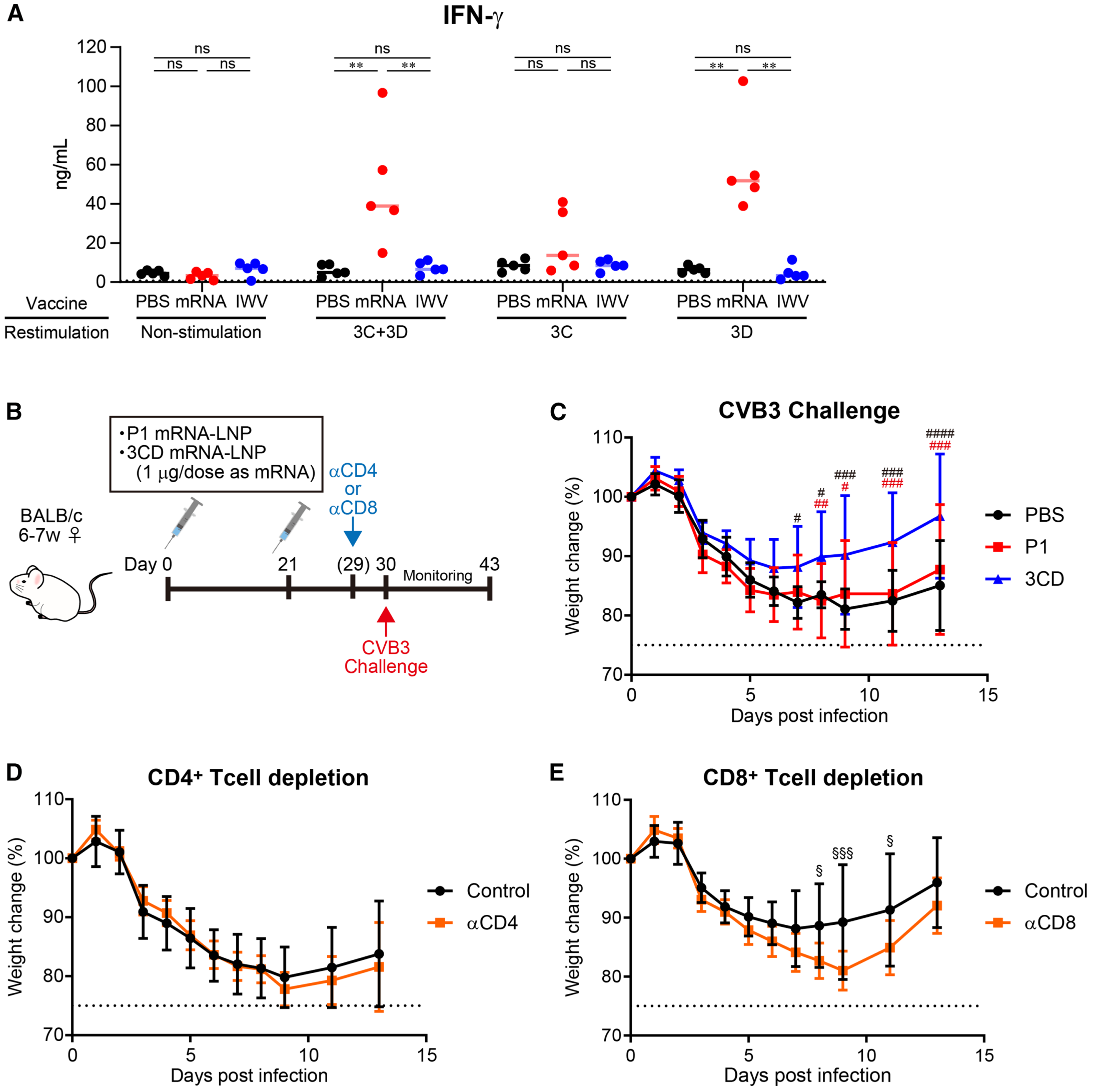

Figure 6. mRNA vaccine expressing VLP induced cellular immunity, contributing to CD8 T$^+$ cell-dependent cross-protection against CVB3

(A) IFN-γ response in splenocytes. The mRNA vaccine (P1:3CD = 1:1 on weight, total 2 μg of mRNA) and IWV (2 μg of protein) immunizations were performed twice before the splenocytes were collected. The splenocytes were cultured in the absence (non-treat) or presence of a mixture of recombinant 3C and 3D, 3C, or 3D protein of EV-D68, and the IFN-γ in the culture supernatants were quantified by ELISA. Points are presented as individual data, and lines are presented as the median. Dotted lines indicate detection limits. (B) Experiment schema for (C–E). The P1 or 3CD mRNA vaccine (1 μg of mRNA per dose) immunizations were performed twice, and CVB3 challenge was started on day 30. For CD4$^+$ or CD8$^+$ T cell depletion, mice vaccinated with 3CD mRNA were administered anti-CD4 (αCD4), anti-CD8 (αCD8), or their isotype control antibodies 1 day before the challenge. (C) The weight change after CVB3 challenge. (D, E) Influence of CD4$^+$ or CD8$^+$ T cell depletion on the efficacy of EV-D68 3CD against CVB3 challenge. (C–E) Data are shown as means ± SD. Dotted line represents the humane endpoint. (A) **$p < 0.01$; ns, not significant as indicated by Tukey's test. (C) #$p < 0.05$; ##$p < 0.01$; ###$p < 0.001$; ####$p < 0.0001$ as indicated by Dunnett's test compared with 3CD. Black symbols show significance of PBS against 3CD, and red symbols show significance of P1 against 3CD. (D, E) §$p < 0.05$; §§§$p < 0.001$; as indicated by Šidák's test compared with isotype control group.

nose alone is insufficient to induce mucosal antibody production. Further investigation is required to elucidate the exact mechanism of nasal IgA induction by intramuscular vaccination. On the contrary, it should be noted that the LNP formulation we used is not appropriate for intranasal vaccines.[61] LNP compositions for further induction of nasal antibodies for intranasal vaccination are still under development and have so far been reported in only a few publications.[62–64] The development of such LNP compositions for intranasal vaccines is also desired in the future.

Although vaccine-induced antibodies are important for preventing viral infections, vaccine-induced CD8$^+$ T cells may also be important for viral clearance. In major respiratory infections such as SARS-CoV-2[65–67] and influenza,[68,69] vaccine-induced CD8$^+$ T cells have

been reported to attenuate disease severity. Additionally, evidence suggests that $CD8^+$ T cells play a role in the clearance of viruses by EV-A71[70] and CVB3.[71] In this study, we showed that EV-D68 3CD-specific $CD8^+$ T cells induced by the mRNA vaccine contributed to cross-protection against other enteroviruses. Sequence homology within the enterovirus genus tends to be higher in the non-structural than in the structural protein region; the sequence homology between EV-D68 (MO strain) and CVB3 (Nancy strain) was higher for 3CD (71.78%; Figure S7) than for P1 (43.79%; Figure S8). In addition, the presence of a class I epitope (CVB3 1765-1774; VLRSGDPRLK) has been reported in the 3D structure of CVB3,[72] and EV-D68 has a similar epitope (VLNPKDPRLK) in 3D. These results suggest that the mRNA vaccine induced $CD8^+$ T cells against non-structural proteins, which could eliminate various enteroviruses.

In the present study, the VP1 mRNA vaccine induced EV-D68-specific IgG, but did not induce neutralizing antibodies against EV-D68. A major neutralizing epitope is present in the VP1 region of enteroviruses.[15] For example, it has been reported that the recombinant VP1 protein of EV-A71 expressed in *E. coli* can induce neutralizing antibodies equivalent to an inactivated whole-particle vaccine of EV-A71.[73,74] Therefore, VP1 mRNA is considered an antigen with the potential to induce neutralizing antibodies. The reason why VP1 mRNA failed to induce neutralizing antibodies is unclear, but our results of the *in vitro* expression study suggest that VP1 expressed by the VP1 mRNA vaccines with secretory signal peptides undergo post-translational modifications such as glycosylation, which are not added to the EV-D68 virus. Such modifications may result in structural changes and masking of the neutralizing epitope.[75] To overcome these challenges, sequence modifications are required in VP1 mRNA vaccines to induce neutralizing antibodies.

This study has some limitations. First, the mRNA vaccine expressed VLP-induced anti-EV-D68 IgA in the nasal wash, but we could not clearly show the contributions of IgA to the neutralizing activity and *in vivo* protection compared with that of IgG in the nasal wash. In addition, 3C protease has been reported for its toxicity, such as disruption of innate immune pathways[76,77] and neurotoxicity,[78] and the 3CD we used in the vaccine may also have the potential risks of these toxicity. The safety of the vaccine was not examined in the current study and needs careful evaluation. Moreover, since we did not have a mouse model of EV-A71 infection, such as the human scavenger receptor class B member 2 (hSCARB-2)-knockin mouse,[79,80] we could not evaluate cross-protection of EV-A71. EV-A71 is also a cause of AFM, and demonstrating that this vaccine provides cross-protection against EV-A71 would be important to establish the vaccine's superiority. Finally, the expected target population for an EV-D68 vaccine is young children because EV-D68-induced respiratory illness and AFM occur predominantly in children; however, data from clinical trials of mRNA vaccination in children are limited compared with those in adults. The SARS-CoV-2 spike mRNA vaccine has been shown to be effective and safe in children aged 5–11 years,[81,82] but the data are still limited especially in children aged 4 years and younger. Continued research on the use of mRNA vaccines in children and the development of basic technologies to improve the efficacy and safety of mRNA vaccines in children are expected.

In conclusion, we established an mRNA vaccine platform against EV-D68. As the formation of VLPs from P1 and 3CD is a common mechanism in enteroviruses, this platform could be applied to other strains of EV-D68 and other enteroviruses, including EV-A71 or coxsackieviruses A6 and A16, which cause hand, foot, and mouth disease, and coxsackievirus group B, which causes aseptic meningitis and pancreatitis. This mRNA vaccine may offer a basis for the development of solutions in overcoming enteroviral infections.

METHODS

Ethics statements

All animal experiments were conducted in accordance with the institutional guidelines of The University of Osaka for the ethical treatment of animals. The protocols were approved by the Animal Care and Use Committee of the Research Institute for Microbial Diseases, The University of Osaka, Japan (protocol number: BIKEN-AP-R01-15-3). All experiments involving EV-D68 were approved by the Institutional Review Board of the Research Institute for Microbial Diseases at The University of Osaka (protocol number: BIKEN-00184-004). All genetic experiments were performed according to the institutional guidelines of The University of Osaka (protocol number BIKEN-04695).

Cells and viruses

Rhabdomyosarcoma (RD-A) cells were kindly provided by Dr. Hiroyuki Shimizu (National Institute of Infectious Diseases, Tokyo, Japan). Vero and HEK293 cells were obtained from the American Type Culture Collection (ATCC, Manassas, Virginia, USA). All the cells were cultured in DMEM (high glucose) supplemented with 10% heat-inactivated fetal bovine serum (FBS), 1% penicillin, and 1% streptomycin at 37°C in a humidified incubator with 5% CO_2, and subculture was performed twice or thrice a week. The EV-D68 strains US/MO/14-18947 (GenBank: KM851225.1), US/IL/14-18952 (GenBank: KM851230.1), and US/KY/14-18953 (GenBank: KM851231.1) used in this study were purchased from ATCC, while EV-D68 1520-Yamagata-2015 strain[36] (GenBank: LC203538.1) and EVD68/Yamagata.JPN/2023-89 strain[37] (GenBank: LC815026.1) were kindly provided by the Department of Microbiology, Yamagata Prefectural Institute of Public Health, Yamagata, Japan. The CVB3 we used was a Nancy strain (GenBank: AJ295194.1). All EV-D68 were propagated in RD-A cells at 33°C, and CVB3 were propagated in Vero cells at 37°C. Unless otherwise stated, DMEM (high glucose) supplemented with 2% FBS, 1% penicillin, and 1% streptomycin was used for the maintenance of all viral cultures. Viral titers were determined using the Karber method[83] and expressed as 50% tissue culture infectious dose ($TCID_{50}$).

Gene

The mRNAs encoding EV-D68 VP1, P1, and 3CD from the US/MO/14-18947 strain (GenBank: KM851225.1) were used. All genes were

optimized for mammalian codon usage. In some experiments, secretory signal peptides derived from human IgGκ chains or IL-2 were added to the N-terminus of EV-D68 VP1. The sequences of the genes used are provided in Table S1.

mRNA synthesis

The template plasmid DNA for mRNA was prepared using the Cloning Kit for mRNA Template (catalog number #6143, Takara Bio, Shiga, Japan) following the manufacturer's instructions. The DNA fragments encoding the VP1, P1, and 3CD of EV-D68 were amplified by PCR using KOD One^R PCR Master Mix (catalog number #KMM-101, TOYOBO, Osaka, Japan) and cloned into a linearized template vector. The template plasmid DNAs were linearized with HindIII-HF restriction enzyme (catalog number #R3104; New England Biolabs, Ipswich, MA, USA) and purified using phenol/chloroform/isoamyl alcohol (25:24:1). The mRNAs were synthesized with Takara IVTpro T7 mRNA Synthesis Kit (catalog number #6144, Takara Bio) following the manufacturer's instructions with the following modifications. Briefly, the reaction mixture (linearized template DNA; 1× transcription buffer; 10 mM ATP, CTP, and GTP,;10 mM N^1-methylpseudouridine 5′-triphosphate [$m^1\Psi$TP] [catalog number #M3544, TCI, Tokyo, Japan]; 8 mM CleanCap Reagent AG [catalog number #N-7113, TriLink, San Diego, USA], and 1× Enzyme Mix) was incubated for 2 h in 37°C. For the firefly luciferase mRNA, we used the Positive Control Template (FLuc) in the kit as template DNA. The products were purified using LiCl precipitation and suspended in nuclease-free water. The dsRNA contaminants were removed using a previously described cellulose-based purification method.[84] Briefly, RNA synthesized by *in vitro* transcription (IVT) was mixed with cellulose suspended in chromatography buffer (10 mM HEPES, 0.1 mM EDTA, 125 mM NaCl, 16% [v/v] ethanol) and shaken vigorously at 37°C for dsRNA to adsorb onto the cellulose. The non-adsorbed RNA was collected and precipitated with 0.3 M sodium acetate and 50% (v/v) 2-propanol. The precipitated mRNA was dissolved in nuclease-free water and stored at −80°C.

RNA electrophoresis

RNA electrophoresis was conducted using DynaMarker RNA High for Easy Electrophoresis (BDL, Tokyo, Japan), following the manufacturer's instructions. The mRNAs (1 μg/lane) were denatured in sample buffer containing formaldehyde and formamide at 65°C for 3 min. The denatured samples were electrophoresed on a 1% agarose gel containing 1× TAE buffer (Tris-Acetate-EDTA buffer; 40 mM Tris, 40 mM acetic acid, 1 mM EDTA) and 0.006% (v/v) MIDORI Green Xtra (Nippon Genetics, Tokyo, Japan). Images were acquired using a FAS-IV imaging system (Nippon Genetics).

LNP preparation

The LNP formulation we used was previously described[85] and composed of SM-102 (catalog number #33474, Cayman Chemical, Ann Arbor, Michigan, USA), 1,2-distearoyl-sn-glycero-3-phosphocholine (DSPC) (catalog number #MC-8080, Yuka Sangyo, Tokyo, Japan), cholesterol (catalog number #C8667, Sigma-Aldrich, Burlington, Massachusetts, USA), and 1,2-dimyristoyl-rac-glycero-3-methylpolyoxyethylene (DMG-PEG)-2000 (catalog number #GM-020, Yuka Sangyo). The lipids were dissolved in ethanol at a molar ratio 50:10:38.5:1.5 for SM-102:DSPC:cholesterol:DMG-PEG-2000. The lipid solution was mixed with mRNA in acetate buffer (6.25 mM, pH 5.0) at a volume ratio of ethanol:water = 3:1 and a total flow ratio of 2 mL/min using NanoAssemblr Ignite (Precision NanoSystems, Vancouver, BC, Canada). The formulation contained mRNA and lipids at an N:P ratio of 5.5. The mixture was quickly diluted with three volumes of MES buffer (20 mM, pH5.5), followed by buffer exchange with Dulbecco's PBS (D-PBS) using an Amicon Ultra-15 Ultracell-PL 100kDa (Merck, Burlington, Massachusetts, USA). The hydrodynamic diameter and zeta potential of the mRNA-LNPs were measured using dynamic light scattering (Zetasizer Nano-ZS; Malvern Panalytical Ltd., Worcestershire, UK). The mRNA encapsulated into LNP was quantified with Quant-it RiboGreen RNA Assay Kit (Invitrogen, Waltham, Massachusetts, USA) following the manufacturer's instructions. For the total mRNA concentration in mRNA-LNP suspension (sum of encapsulated and free RNA), the mRNA-LNP was disrupted with 0.2% (v/v) polyoxyethylene(10) octylphenyl ether (WAKO, Osaka, Japan) before assay.

Preparation of IWV vaccines

We prepared EV-D68 as previously described.[34] The EV-D68 MO strain was propagated in RD-A cells in VP-SFM (1×) (ThermoFisher Scientific), supplemented with 4 mM L-alanyl-L-glutamine and incubated at 33°C, 5% CO_2 for 2 days. For virus inactivation, 0.05% β-propiolactone (WAKO) was added to the culture supernatant before incubation at 4°C for 24 h. β-propiolactone was hydrolyzed for 2 h at 37°C. The resulting supernatant underwent purification through a 20% sucrose cushion (Beckman SW32Ti rotor, 141,000 × *g* for 3 h at 4°C) and 10%–40% continuous sucrose gradient ultracentrifugation (Beckman SW32Ti rotor, 130,000 × *g* for 4 h at 4°C). Fractions primarily containing full particles were concentrated using Amicon Ultra Centrifugal Filters (100 kDa MWCO).

In vitro expression

HEK293 cells were seeded on 24-well plates at 2.0 × 10^5 cells/well and incubated for 1 day at 37°C and 5% CO_2. mRNA was introduced using Lipofectamine MessengerMAX (Thermo Fisher Scientific, Waltham, Massachusetts, USA) following the manufacturer's instructions. Briefly, 0.5 μg mRNA and 0.75 μL of Lipofectamine reagent per well were mixed and dropped onto cells. After a 24-h incubation, culture supernatant was harvested, and cell lysates were collected using RIPA buffer (50 mM Tris, 150 mM NaCl, 1% [v/v] Nonidet P40, 0.5% [w/v] sodium deoxycholate, and 0.1% [w/v] SDS). For SDS-PAGE, the samples were mixed with a sample buffer solution containing 2-mercaptoethanol, and then heated at 95°C for 5 min prior to loading onto a 10% polyacrylamide gel. Following electrophoresis, the proteins were transferred onto a polyvinylidene fluoride membrane (Bio-Rad Laboratories, Inc., Hercules, CA, USA). Subsequently, the membrane was blocked using 5% (w/v) skim milk in PBS supplemented with 0.5% (v/v) Tween 20 (PBS-T), followed by

sequential incubation with rabbit polyclonal anti-EV-D68 VP1 (catalog number #GTX132313, dilution: 1/3,000, GeneTex, Irvine, CA, USA) as primary antibody and anti-rabbit IgG secondary antibody with horseradish peroxidase (HRP) (catalog number #458, dilution: 1/5,000, Medical & Biological Laboratories, Tokyo, Japan). Signals were detected using the ChemiDoc Touch Imaging System (Bio-Rad Laboratories, Inc.). To confirm VLP formation by sucrose density gradient, 20 μg mRNA was transfected into HEK293 cells seeded on 150-mm plates at 8×10^6 cells/dish following the procedure described above. The culture supernatant underwent fractionation through a 20% sucrose cushion (Beckman SW32Ti rotor, 141,000 × *g* for 3 h at 4°C) and 10%–40% continuous sucrose gradient ultracentrifugation (Beckman SW32Ti rotor, 130,000 × *g* for 4 h at 4°C), and the resulting fractions were subjected to western blotting using anti-VP1 antibodies.

Mice

BALB/c mice were obtained from SLC (Hamamatsu, Shizuoka, Japan). The mice were housed in a room with a 12/12-h light/dark cycle (lights on, 8:00 a.m.; lights off, 8:00 p.m.) and *ad libitum* feeding. The mice were anesthetized via intraperitoneal injection of a mixture of three drugs: 0.3 mg/kg medetomidine (Nippon Zenyaku Kogyo Co., Ltd., Tokyo, Japan) for sedation, analgesic effect, and muscle relaxation; 4 mg/kg midazoram (Maruishi Pharmaceutical Co., Ltd., Osaka, Japan) for sedation; and 5 mg/kg butorphanol (Meiji Animal Health Co., Ltd., Kumamoto, Japan) for analgesia. The mice were euthanized via CO_2 inhalation.

In vivo expression

The mRNA vaccine expressing VLP (P1:3CD = 1:1 by weight, 2 μg of total mRNA) was injected intramuscularly into mice under anesthesia. Six hours after injection, the mice were sacrificed, and the muscles were harvested. The muscle was homogenized in RIPA buffer using a μT-12 bead crusher (TAITEC, Saitama, Japan) and centrifuged at 2,100 × *g* for 10 min. The supernatant was then used for western blot analysis.

Vaccination

For intramuscular vaccination, mice (6–7 weeks old, female) were anesthetized, and mRNA-LNPs (2, 0.4, 0.08, 0.016, or 0.0032 μg of mRNA) or IWV vaccines (10, 2, 0.4, 0.08, or 0.016 μg of protein) were injected into the thigh muscle on days 0 and 21 (50 μL per dose). Blood, nasal wash, BALF, and nasal tissue samples were collected on day 28, and the spleen was collected on day 35. For the challenge experiments, EV-D68 was administered on day 31 and CVB3 was administered on day 30.

Detection of EV-D68-specific antibodies

To identify MO strain-specific IgG, IgG1, IgG2a, IgG2b, and IgA, ELISA plates (Corning Incorporated, Corning, NY, USA) were coated with 0.6 μg/mL IWV in carbonate buffer at 4°C. When evaluating antibodies in nasal wash and BALF, plates were coated with 6 μg/mL IWV. After coating, the plates were treated with 1% Block Ace (DS Pharma Biomedical, Osaka, Japan) for 1 h at 25°C. Plasma, nasal washes, and BALF samples underwent serial dilution with 0.4% Block Ace, and these dilutions were applied to the antigen-coated plates. After a 2-h incubation at room temperature, the plates were exposed to HRP-conjugated goat anti-mouse IgG (catalog number #1030-05, dilution: 1/5,000, SouthernBiotech, Birmingham, AL, USA), IgG1 (catalog number #1073-05, dilution: 1/5,000, SouthernBiotech), IgG2a (catalog number #1083-05, dilution: 1/5,000, SouthernBiotech), IgG2b (catalog number #1093-05, dilution: 1/5,000, SouthernBiotech), and IgA (catalog number #1040-05, dilution: 1/5,000, SouthernBiotech) for 1 h at room temperature. The colorimetric reaction was initiated using tetramethylbenzidine, halted with 2 N H_2SO_4, and assessed at $OD_{450–530}$ nm on a microplate reader (PowerWave HT; Bio-Tek Instruments, Inc., USA). Regression analysis of the sample dilution factor and $OD_{450\text{-}530}$ value was executed utilizing 4-parameter logistic analysis, and the dilution factor corresponding to an $OD_{450\text{-}530}$ of 0.2 was determined as the endpoint titer.

ELISpot assay

The noses of vaccinated mice were harvested under anesthesia. Internal nasal tissue was scraped using a Horkman two-headed sharp spoon, suspended in D-PBS, and pelleted. The supernatant was collected, and the pellet was suspended in RPMI containing 0.25 mg/mL Liberase TL (Roche Diagnostics, Mannheim, Germany) and 100 U/mL DNase I (WAKO) and digested with shaking at 37°C for 45 min. The tissue was ground using a plunger and filtered through a 70-μm cell strainer. The supernatant and digested cells were pooled and hemolyzed using red blood cell lysis buffer (8.3 g/L NH_4Cl and 10 mM Tris-HCl at pH 7.5). The cells were pelleted down and suspended in RPMI1640 medium supplemented with 10% FBS, 50 μM 2-mercaptoethanol, 1% penicillin, and 1% streptomycin. The cell suspension was added into Multiscreen plate (catalog number #MSIPS4W10, Merck) coated with IWV (0.6 μg/mL in carbonate buffer) and treated with RPMI1640 medium supplemented with 5% FBS for blocking. After incubation at 5% CO_2 at 37°C for 4 h, the plates were washed with PBS-T and incubated with HRP-conjugated goat anti-mouse IgG (catalog number #1030-05, dilution: 1/5,000, SouthernBiotech) or IgA (catalog number #1040-05, dilution: 1/5,000, SouthernBiotech) for 1 h at room temperature. To develop color, ELISpot substrate (Mabtech, Nacka Strand, Sweden) was added at 100 μL per well for 10 min. After washing, the plates were photographed using an ImmunoSpot S5 Micro Analyzer (Cellular Technology Limited, Shaker Heights, OH, USA). The dots in each well were counted and multiplied by the dilution factor of the cell suspension to calculate the number of EV-D68-specific ASCs per tissue sample.

Neutralization assay

Titers of neutralizing antibodies against EV-D68 were determined using microneutralization assays. Plasma samples underwent heat inactivation at 56°C for 30 min, while BALF and nasal wash samples were not inactivated. Subsequently, the samples were subjected to serial 2-fold dilutions in DMEM supplemented with 2% FBS, 1% penicillin, and 1% streptomycin. Following dilution, the plasma samples

were exposed to 100 $TCID_{50}$/well of EV-D68, and the BALF and nasal wash samples were exposed to 25 $TCID_{50}$/well of EV-D68 at 37°C for 1 h. RD-A cells were seeded into each well at a density of 1.5×10^4 cells/well and incubated at 33°C with 5% CO_2 for 7 days to enable EV-D68 infection. Cytopathic effects (CPE) were monitored in the cells, and neutralizing titers were determined as the highest serum dilutions capable of completely preventing CPE.

Viral challenge

On day 10 post-boost immunization, the vaccinated mice were intranasally challenged with 5.0×10^6 $TCID_{50}$ of EV-D68 in a total volume of 20 μL in PBS (10 μL per nostril) under anesthesia. At 12 h post-infection, samples from the nasal turbinates and lungs were collected in screw-cap tubes containing 4.0-mm stainless beads (TAITEC) and mechanically homogenized by a μT-12 beads crusher (TAITEC) in 1 mL DMEM. The resulting lysates were then centrifuged at 10,000 × *g* for 3 min at 4°C, and viral titers in the supernatants were assessed using the $TCID_{50}$ assay. For the CVB3 challenge, the vaccinated mice were intraperitoneally challenged with 7.0×10^5 $TCID_{50}$ of CVB3 in a total volume of 200 μL in DMEM on day 9 post-boost immunization. For depletion of $CD4^+$ T cells, 100 μg of anti-mouse CD4 antibody (catalog number #BE0003-1, clone: GK1.5, Bio X Cell, West Lebanon, NH, USA) or IgG2b isotype control (catalog number #BE0090, clone: LTF-2, Bio X Cell) was administered intraperitoneally 1 day before the challenge. For $CD8^+$ T cell depletion, 100 μg of anti-mouse CD8β antibody (catalog number #BE0223, clone: 53-5.8, Bio X Cell) or IgG1 isotype control (catalog number #BE0088, clone: HRPN, Bio X Cell) was administered intraperitoneally 1 day before the challenge. Weight changes were monitored daily from the day of challenge. The humane endpoint was set at 25% body weight loss relative to that at day 0. Mice that had reached the humane endpoint or survived for 13 days after the challenge were euthanized.

Passive antisera transfer experiments

Naive 1-day-old BALB/c mice were intraperitoneally administered 50 μL of vaccinated sera (diluted to 1:4 with DMEM supplemented with 2% FBS, 1% penicillin, and 1% streptomycin) obtained from adult mice that had been immunized twice. The following day, mice were intraperitoneally challenged with 3.2×10^6 $TCID_{50}$ of EV-D68 (100 μL). Survival and paralysis of infected mice were assessed daily for 14 days following serum transfer. Mice that survived for 14 days after serum transfer were euthanized. To quantify the viral titer in spinal cord, the spinal cord was harvested 4 days after infection and the viral titers were quantified using $TCID_{50}$ assay as described above.

IFN-γ and IL-4 quantification from splenocyte

Fourteen days after booster immunization, the spleen was harvested under anesthesia. It was mashed, strained through a 70-μm cell strainer, and hemolyzed using ACT lysis buffer. Subsequently, splenocytes (1×10^6 cells) were restimulated with 10 μg/mL IWV or recombinant 3C and 3D protein over a period of 60 h at 37°C in 96-well U-bottom plates, and the culture supernatant were collected. IFN-γ and IL-4 were quantified using ELISA MAX Standard Set Mouse IFN-γ or IL-4 (BioLegend, San Diego, CA, USA) following the manufacturer's instructions.

Luciferase activity quantification

To analyze firefly luciferase mRNA expression in tissue organs, mice (6–7 weeks old, female) were administered with mRNA-LNP of firefly luciferase (3 μg mRNA per mice) in a total volume of 50 μL for intramuscular administration or 20 μL (10 mL to each nostril) for intranasal administration. Six hours after inoculation, the muscle, nasal turbinate, lungs, liver, and spleen were harvested under anesthesia and homogenized in 1 mL of D-PBS. Luciferase activity in the tissue homogenate was quantified using a plate-based assay with the Bright-Glo Luciferase Assay System (Promega, Madison, Wisconsin, USA). Luminescence was standardized according to the tissue weight.

Statistical analyses

Statistical analyses were performed using Prism 10 software version 10.3.1 (GraphPad Software, San Diego, CA, USA). Significant differences were determined using Tukey's test. Statistical significance was set at $p < 0.05$. Each experiment was performed at least twice.

DATA AND CODE AVAILABILITY

All data are included in the paper or the supplemental information. Please contact the lead researcher Yasuo Yoshioka (y-yoshioka@biken.osaka-u.ac.jp) for additional information or requests concerning resources and reagents.

ACKNOWLEDGMENTS

We thank Dr. Katsumi Mizuta (Department of Microbiology, Yamagata Prefectural Institute of Public Health) for providing EV-D68 1520-Yamagata-2015 and EVD68/Yamagata.JPN/2023-89 strains. We thank Dr. Hirotaka Ebina and Mr. Takafumi Noguchi (Virus Vaccine Group, BIKEN Innovative Vaccine Research Alliance Laboratories, Institute for Open and Transdisciplinary Research Initiatives, The University of Osaka) for providing CVB3 virus. We thank Dr. Shuhei Taguwa and Ms. Chizuko Kominami (Center for Infectious Disease Education & Research/Research Institute for Microbial Diseases, The University of Osaka) for useful technical advice. We thank Dr. Hidesato Ogawa and Ms. Akiho Yoshida (The Research Foundation for Microbial Diseases of Osaka University) for help with manuscript revision. This research received funding from several sources, including the Japan Society for the Promotion of Science (JSPS KAKENHI grant numbers: 23K27343 and 24K22020 to Y.Y.) and the Japan Agency for Medical Research and Development (AMED grant number: JP223fa627002 to Y.Y.). It also received support from All-Osaka U Research under the Nippon Foundation–The University of Osaka Project for Infectious Disease Project (to Y.Y.) and the Research Foundation for Microbial Diseases of Osaka University (BIKEN).

AUTHOR CONTRIBUTIONS

Y.K. and Y.Y. designed the experiments. Y.K. performed the experiments and analyzed and interpreted the data. K.S., C.K.-N., and T.H. contributed to the experimental design and edited the manuscript. Y.K. and Y.Y. drafted the manuscript. Y.Y. supervised the study. All the authors have read and agreed to the published version of the manuscript.

DECLARATION OF INTERESTS

Y.K., C.K.-N., and Y.Y. were employed by the Research Foundation for Microbial Diseases of Osaka University.

SUPPLEMENTAL INFORMATION

Supplemental information can be found online at https://doi.org/10.1016/j.omtn.2025.102731.

REFERENCES

1. Schieble, J.H., Fox, V.L., and Lennette, E.H. (1967). A probable new human picornavirus associated with respiratory diseases. Am. J. Epidemiol. *85*, 297–310. https://doi.org/10.1093/oxfordjournals.aje.a120693.
2. Benschop, K.S., Albert, J., Anton, A., Andrés, C., Aranzamendi, M., Armannsdóttir, B., Bailly, J.-L., Baldanti, F., Baldvinsdóttir, G.E., Beard, S., et al. (2021). Re-emergence of enterovirus D68 in Europe after easing the COVID-19 lockdown, September 2021. Euro Surveill. *26*, 2100998. https://doi.org/10.2807/1560-7917.ES.2021.26.45.2100998.
3. Holm-Hansen, C.C., Midgley, S.E., and Fischer, T.K. (2016). Global emergence of enterovirus D68: a systematic review. Lancet Infect. Dis. *16*, e64–e75. https://doi.org/10.1016/S1473-3099(15)00543-5.
4. Messacar, K., and Tyler, K.L. (2019). Enterovirus D68-Associated Acute Flaccid Myelitis: Rising to the Clinical and Research Challenges. JAMA *321*, 831–832. https://doi.org/10.1001/jama.2019.1016.
5. McLaren, N., Lopez, A., Kidd, S., Zhang, J.X., Nix, W.A., Link-Gelles, R., Lee, A., and Routh, J.A. (2020). Characteristics of patients with acute flaccid myelitis, United States, 2015-2018. Emerg. Infect. Dis. *26*, 212–219. https://doi.org/10.3201/eid2602.191453.
6. Baggen, J., Thibaut, H.J., Strating, J.R.P.M., and van Kuppeveld, F.J.M. (2018). The life cycle of non-polio enteroviruses and how to target it. Nat. Rev. Microbiol. *16*, 368–381. https://doi.org/10.1038/s41579-018-0005-4.
7. Elrick, M.J., Pekosz, A., and Duggal, P. (2021). Enterovirus D68 molecular and cellular biology and pathogenesis. J. Biol. Chem. *296*, 100317. https://doi.org/10.1016/j.jbc.2021.100317.
8. Liu, Y., Sheng, J., Baggen, J., Meng, G., Xiao, C., Thibaut, H.J., van Kuppeveld, F.J.M., and Rossmann, M.G. (2015). Sialic acid-dependent cell entry of human enterovirus D68. Nat. Commun. *6*, 8865. https://doi.org/10.1038/ncomms9865.
9. Wei, W., Guo, H., Chang, J., Yu, Y., Liu, G., Zhang, N., Willard, S.H., Zheng, S., and Yu, X.-F. (2016). ICAM-5/telencephalin is a functional entry receptor for Enterovirus D68. Cell Host Microbe *20*, 631–641. https://doi.org/10.1016/j.chom.2016.09.013.
10. Baggen, J., Liu, Y., Lyoo, H., van Vliet, A.L.W., Wahedi, M., de Bruin, J.W., Roberts, R.W., Overduin, P., Meijer, A., Rossmann, M.G., et al. (2019). Bypassing pan-enterovirus host factor PLA2G16. Nat. Commun. *10*, 3171. https://doi.org/10.1038/s41467-019-11256-z.
11. Liu, X., Li, H., Li, Z., Gao, D., Zhou, J., Ni, F., Yu, Q., Huang, Y., Tang, Y., Xue, L., et al. (2025). MFSD6 is an entry receptor for respiratory enterovirus D68. Cell Host Microbe *33*, 267–278.e4. https://doi.org/10.1016/j.chom.2024.12.015.
12. Varanese, L., Xu, L., Peters, C.E., Pintilie, G., Roberts, D.S., Raj, S., Liu, M., Ooi, Y.S., Diep, J., Qiao, W., et al. (2025). MFSD6 is an entry receptor for enterovirus D68. Nature *641*, 1268–1275. https://doi.org/10.1038/s41586-025-08908-0.
13. Premanand, B., Kiener, T.K., Meng, T., Tan, Y.R., Jia, Q., Chow, V.T.K., and Kwang, J. (2012). Induction of protective immune responses against EV71 in mice by baculovirus encoding a novel expression cassette for capsid protein VP1. Antiviral Res. *95*, 311–315. https://doi.org/10.1016/j.antiviral.2012.05.017.
14. Yuan, J., Tang, X., Yin, K., Tian, J., Rui, K., Ma, J., Mao, C., Chen, J., Lu, L., Xu, H., and Wang, S. (2015). GITRL as a genetic adjuvant enhances enterovirus 71 VP1 DNA vaccine immunogenicity. Immunol. Res. *62*, 81–88. https://doi.org/10.1007/s12026-015-8637-1.
15. Zheng, H., Wang, J., Li, B., Guo, L., Li, H., Song, J., Yang, Z., Li, H., Fan, H., Huang, X., et al. (2018). A Novel Neutralizing Antibody Specific to the DE Loop of VP1 Can Inhibit EV-D68 Infection in Mice. J. Immunol. *201*, 2557–2569. https://doi.org/10.4049/jimmunol.1800655.
16. Hodcroft, E.B., Dyrdak, R., Andrés, C., Egli, A., Reist, J., García Martínez de Artola, D., Alcoba-Flórez, J., Niesters, H.G.M., Antón, A., Poelman, R., et al. (2022). Evolution, geographic spreading, and demographic distribution of Enterovirus D68. PLoS Pathog. *18*, e1010515. https://doi.org/10.1371/journal.ppat.1010515.
17. Dai, W., Li, X., Liu, Z., and Zhang, C. (2023). Identification of four neutralizing antigenic sites on the enterovirus D68 capsid. J. Virol. *97*, e0160023. https://doi.org/10.1128/jvi.01600-23.
18. Chong, P., Liu, C.-C., Chow, Y.-H., Chou, A.-H., and Klein, M. (2015). Review of enterovirus 71 vaccines. Clin. Infect. Dis. *60*, 797–803. https://doi.org/10.1093/cid/ciu852.
19. Hird, T.R., and Grassly, N.C. (2012). Systematic review of mucosal immunity induced by oral and inactivated poliovirus vaccines against virus shedding following oral poliovirus challenge. PLoS Pathog. *8*, e1002599. https://doi.org/10.1371/journal.ppat.1002599.
20. Burns, C.C., Diop, O.M., Sutter, R.W., and Kew, O.M. (2014). Vaccine-derived polioviruses. J. Infect. Dis. *210*, S283–S293. https://doi.org/10.1093/infdis/jiu295.
21. Minor, P.D. (2004). Polio eradication, cessation of vaccination and re-emergence of disease. Nat. Rev. Microbiol. *2*, 473–482. https://doi.org/10.1038/nrmicro906.
22. Li, M.-L., Shih, S.-R., Tolbert, B.S., and Brewer, G. (2021). Enterovirus A71 vaccines. Vaccines (Basel) *9*, 199. https://doi.org/10.3390/vaccines9030199.
23. Dai, W., Zhang, C., Zhang, X., Xiong, P., Liu, Q., Gong, S., Geng, L., Zhou, D., and Huang, Z. (2018). A virus-like particle vaccine confers protection against enterovirus D68 lethal challenge in mice. Vaccine *36*, 653–659. https://doi.org/10.1016/j.vaccine.2017.12.057.
24. Krug, P.W., Wang, L., Shi, W., Kong, W.-P., Moss, D.L., Yang, E.S., Fisher, B.E., Morabito, K.M., Mascola, J.R., Kanekiyo, M., et al. (2023). EV-D68 virus-like particle vaccines elicit cross-clade neutralizing antibodies that inhibit infection and block dissemination. Sci. Adv. *9*, eadg6076. https://doi.org/10.1126/sciadv.adg6076.
25. Li, X., Xu, X., Li, J., Lu, H., Wang, C., Wang, R., Xiao, J., Liu, Y., Song, Y., Song, J., et al. (2024). Preparation and immunoprotective effects of a virus-like particle candidate vaccine of the dominant epidemic D3 genotype coxsackievirus A6 in China. Biosaf. Health *6*, 28–34. https://doi.org/10.1016/j.bsheal.2023.11.001.
26. Hou, X., Zaks, T., Langer, R., and Dong, Y. (2021). Lipid nanoparticles for mRNA delivery. Nat. Rev. Mater. *6*, 1078–1094. https://doi.org/10.1038/s41578-021-00358-0.
27. Chaudhary, N., Weissman, D., and Whitehead, K.A. (2021). mRNA vaccines for infectious diseases: principles, delivery and clinical translation. Nat. Rev. Drug Discov. *20*, 817–838. https://doi.org/10.1038/s41573-021-00283-5.
28. Wilson, E., Goswami, J., Baqui, A.H., Doreski, P.A., Perez-Marc, G., Zaman, K., Monroy, J., Duncan, C.J.A., Ujiie, M., Rämet, M., et al. (2023). Efficacy and safety of an mRNA-based RSV PreF vaccine in older adults. N. Engl. J. Med. *389*, 2233–2244. https://doi.org/10.1056/NEJMoa2307079.
29. Ananworanich, J., Lee, I.T., Ensz, D., Carmona, L., Schaefers, K., Avanesov, A., Stadlbauer, D., Choi, A., Pucci, A., McGrath, S., et al. (2025). Safety and immunogenicity of mRNA-1010, an investigational seasonal influenza vaccine, in healthy adults: Final results from a phase 1/2 randomized trial. J. Infect. Dis. *231*, e113–e122. https://doi.org/10.1093/infdis/jiae329.
30. Martin, T.M., Robinson, S.T., and Huang, Y. (2023). Discovery medicine - the HVTN's iterative approach to developing an HIV-1 broadly neutralizing vaccine. Curr. Opin. HIV AIDS *18*, 290–299. https://doi.org/10.1097/COH.0000000000000821.
31. Hu, X., Karthigeyan, K.P., Herbek, S., Valencia, S.M., Jenks, J.A., Webster, H., Miller, I.G., Connors, M., Pollara, J., Andy, C., et al. (2023). Human Cytomegalovirus mRNA-1647 vaccine candidate elicits potent and broad neutralization and higher antibody-dependent cellular cytotoxicity responses than that of the partially effective gB/MF59 vaccine. Preprint at medRxiv. https://doi.org/10.1101/2023.08.02.23293386.
32. Bollman, B., Nunna, N., Bahl, K., Hsiao, C.J., Bennett, H., Butler, S., Foreman, B., Burgomaster, K.E., Aleshnick, M., Kong, W.-P., et al. (2023). An optimized messenger RNA vaccine candidate protects non-human primates from Zika virus infection. NPJ Vaccines *8*, 58. https://doi.org/10.1038/s41541-023-00656-4.
33. Essink, B., Chu, L., Seger, W., Barranco, E., Le Cam, N., Bennett, H., Faughnan, V., Pajon, R., Paila, Y.D., Bollman, B., et al. (2023). The safety and immunogenicity of two Zika virus mRNA vaccine candidates in healthy flavivirus baseline seropositive and seronegative adults: the results of two randomised, placebo-controlled, dose-ranging, phase 1 clinical trials. Lancet Infect. Dis. *23*, 621–633. https://doi.org/10.1016/S1473-3099(22)00764-2.
34. Senpuku, K., Kataoka-Nakamura, C., Kunishima, Y., Hirai, T., and Yoshioka, Y. (2024). An inactivated whole-virion vaccine for Enterovirus D68 adjuvanted with

CpG ODN or AddaVax elicits potent protective immunity in mice. Vaccine *42*, 2463–2474. https://doi.org/10.1016/j.vaccine.2024.03.016.

35. Chandler, T.L., Yang, A., Otero, C.E., Permar, S.R., and Caddy, S.L. (2023). Protective mechanisms of nonneutralizing antiviral antibodies. PLoS Pathog. *19*, e1011670. https://doi.org/10.1371/journal.ppat.1011670.

36. Itagaki, T., Aoki, Y., Matoba, Y., Tanaka, S., Ikeda, T., Mizuta, K., and Matsuzaki, Y. (2018). Clinical characteristics of children infected with enterovirus D68 in an outpatient clinic and the association with bronchial asthma. Infect. Dis. (Lond.) *50*, 303–312. https://doi.org/10.1080/23744235.2017.1400176.

37. Itagaki, T., Komabayashi, K., Sasaki, M., Ogawa, N., Seto, J., Aoki, Y., Ikeda, T., Matsuzaki, Y., and Mizuta, K. (2024). Seroprevalence of enterovirus D68 in Yamagata, Japan, between 1976 and 2019. J. Med. Virol. *96*, e29947. https://doi.org/10.1002/jmv.29947.

38. Yuan, J., Shen, L., Wu, J., Zou, X., Gu, J., Chen, J., and Mao, L. (2018). Enterovirus A71 Proteins: Structure and Function. Front. Microbiol. *9*, 286. https://doi.org/10.3389/fmicb.2018.00286.

39. Wang, S.H., Wang, K., Zhao, K., Hua, S.C., and Du, J. (2020). The Structure, Function, and Mechanisms of Action of Enterovirus Non-structural Protein 2C. Front. Microbiol. *11*, 615965. https://doi.org/10.3389/fmicb.2020.615965.

40. Tsou, Y.L., Lin, Y.W., Shao, H.Y., Yu, S.L., Wu, S.R., Lin, H.Y., Liu, C.C., Huang, C., Chong, P., and Chow, Y.H. (2015). Recombinant Adeno-Vaccine Expressing Enterovirus 71-Like Particles against Hand, Foot, and Mouth Disease. PLoS Negl. Trop. Dis. *9*, e0003692. https://doi.org/10.1371/journal.pntd.0003692.

41. Warner, N.L., Archer, J., Park, S., Singh, G., McFadden, K.M., Kimura, T., Nicholes, K., Simpson, A., Kaelber, J.T., Hawman, D.W., et al. (2024). A self-amplifying RNA vaccine prevents enterovirus D68 infection and disease in preclinical models. Sci. Transl. Med. *16*, eadi1625. https://doi.org/10.1126/scitranslmed.adi1625.

42. Hu, Y.-C., Hsu, J.T.-A., Huang, J.-H., Ho, M.-S., and Ho, Y.-C. (2003). Formation of enterovirus-like particle aggregates by recombinant baculoviruses co-expressing P1 and 3CD in insect cells. Biotechnol. Lett. *25*, 919–925. https://doi.org/10.1023/a:1024071514438.

43. Lampinen, V., Gröhn, S., Lehmler, N., Jartti, M., Hytönen, V.P., Schubert, M., and Hankaniemi, M.M. (2024). Production of norovirus-rotavirus-and enterovirus-like particles in insect cells is simplified by plasmid-based expression. Sci. Rep. *14*, 14874. https://doi.org/10.1038/s41598-024-65316-6.

44. Kim, W., Zhou, J.Q., Horvath, S.C., Schmitz, A.J., Sturtz, A.J., Lei, T., Liu, Z., Kalaidina, E., Thapa, M., Alsoussi, W.B., et al. (2022). Germinal centre-driven maturation of B cell response to mRNA vaccination. Nature *604*, 141–145. https://doi.org/10.1038/s41586-022-04527-1.

45. Röltgen, K., Nielsen, S.C.A., Silva, O., Younes, S.F., Zaslavsky, M., Costales, C., Yang, F., Wirz, O.F., Solis, D., Hoh, R.A., et al. (2022). Immune imprinting, breadth of variant recognition, and germinal center response in human SARS-CoV-2 infection and vaccination. Cell *185*, 1025–1040.e14. https://doi.org/10.1016/j.cell.2022.01.018.

46. Provine, N.M., and Klenerman, P. (2023). Adenovirus vector and mRNA vaccines: Mechanisms regulating their immunogenicity. Eur. J. Immunol. *53*, e2250022. https://doi.org/10.1002/eji.202250022.

47. Neutra, M.R., and Kozlowski, P.A. (2006). Mucosal vaccines: the promise and the challenge. Nat. Rev. Immunol. *6*, 148–158. https://doi.org/10.1038/nri1777.

48. Asahi, Y., Yoshikawa, T., Watanabe, I., Iwasaki, T., Hasegawa, H., Sato, Y., Shimada, S.-I., Nanno, M., Matsuoka, Y., Ohwaki, M., et al. (2002). Protection against influenza virus infection in polymeric Ig receptor knockout mice immunized intranasally with adjuvant-combined vaccines. J. Immunol. *168*, 2930–2938. https://doi.org/10.4049/jimmunol.168.6.2930.

49. Asahi-Ozaki, Y., Yoshikawa, T., Iwakura, Y., Suzuki, Y., Tamura, S.-I., Kurata, T., and Sata, T. (2004). Secretory IgA antibodies provide cross-protection against infection with different strains of influenza B virus. J. Med. Virol. *74*, 328–335. https://doi.org/10.1002/jmv.20173.

50. Umemoto, S., Nakahashi-Ouchida, R., Yuki, Y., Kurokawa, S., Machita, T., Uchida, Y., Mori, H., Yamanoue, T., Shibata, T., Sawada, S.-I., et al. (2023). Cationic-nanogel nasal vaccine containing the ectodomain of RSV-small hydrophobic protein induces protective immunity in rodents. NPJ Vaccines *8*, 106. https://doi.org/10.1038/s41541-023-00700-3.

51. Hemmi, T., Ainai, A., Hashiguchi, T., Tobiume, M., Kanno, T., Iwata-Yoshikawa, N., Iida, S., Sato, Y., Miyamoto, S., Ueno, A., et al. (2022). Intranasal vaccination induced cross-protective secretory IgA antibodies against SARS-CoV-2 variants with reducing the potential risk of lung eosinophilic immunopathology. Vaccine *40*, 5892–5903. https://doi.org/10.1016/j.vaccine.2022.08.049.

52. Tokunoh, N., Tamiya, S., Watanabe, M., Okamoto, T., Anindita, J., Tanaka, H., Ono, C., Hirai, T., Akita, H., Matsuura, Y., and Yoshioka, Y. (2023). A nasal vaccine with inactivated whole-virion elicits protective mucosal immunity against SARS-CoV-2 in mice. Front. Immunol. *14*, 1224634. https://doi.org/10.3389/fimmu.2023.1224634.

53. O'Ryan, M., Bandyopadhyay, A.S., Villena, R., Espinoza, M., Novoa, J., Weldon, W. C., Oberste, M.S., Self, S., Borate, B.R., Asturias, E.J., et al. (2015). Inactivated poliovirus vaccine given alone or in a sequential schedule with bivalent oral poliovirus vaccine in Chilean infants: a randomised, controlled, open-label, phase 4, non-inferiority study. Lancet Infect. Dis. *15*, 1273–1282. https://doi.org/10.1016/S1473-3099(15)00219-4.

54. Brickley, E.B., Wieland-Alter, W., Connor, R.I., Ackerman, M.E., Boesch, A.W., Arita, M., Weldon, W.C., O'Ryan, M.G., Bandyopadhyay, A.S., and Wright, P.F. (2018). Intestinal immunity to Poliovirus following sequential trivalent inactivated polio vaccine/bivalent oral polio vaccine and trivalent inactivated polio vaccine-only immunization schedules: Analysis of an open-label, randomized, controlled trial in Chilean infants. Clin. Infect. Dis. *67*, S42–S50. https://doi.org/10.1093/cid/ciy603.

55. McGhee, J.R. (2011). A mucosal gateway for vaccines. Nat. Biotechnol. *29*, 136–138. https://doi.org/10.1038/nbt.1766.

56. Aaen, K.H., Anthi, A.K., Sandlie, I., Nilsen, J., Mester, S., and Andersen, J.T. (2021). The neonatal Fc receptor in mucosal immune regulation. Scand. J. Immunol. *93*, e13017. https://doi.org/10.1111/sji.13017.

57. Cao, K.T., Cobos-Uribe, C., Knight, N., Jonnalagadda, R., Robinette, C., Jaspers, I., and Rebuli, M.E. (2023). SARS-CoV-2 mRNA vaccination induces an intranasal mucosal response characterized by neutralizing antibodies. J. Allergy Clin. Immunol. Glob. *2*, 100129. https://doi.org/10.1016/j.jacig.2023.100129.

58. Bladh, O., Aguilera, K., Marking, U., Kihlgren, M., Greilert Norin, N., Smed-Sörensen, A., Sällberg Chen, M., Klingström, J., Blom, K., Russell, M.W., et al. (2024). Comparison of SARS-CoV-2 spike-specific IgA and IgG in nasal secretions, saliva and serum. Front. Immunol. *15*, 1346749. https://doi.org/10.3389/fimmu.2024.1346749.

59. Sano, K., Bhavsar, D., Singh, G., Floda, D., Srivastava, K., Gleason, C., PARIS Study Group, Carreño, J.M., Simon, V., and Krammer, F. (2022). SARS-CoV-2 vaccination induces mucosal antibody responses in previously infected individuals. Nat. Commun. *13*, 5135. https://doi.org/10.1038/s41467-022-32389-8.

60. Puhach, O., Bellon, M., Adea, K., Bekliz, M., Hosszu-Fellous, K., Sattonnet, P., Hulo, N., Kaiser, L., Eckerle, I., and Meyer, B. (2023). SARS-CoV-2 convalescence and hybrid immunity elicits mucosal immune responses. EBioMedicine *98*, 104893. https://doi.org/10.1016/j.ebiom.2023.104893.

61. Vu, M.N., Pilapitiya, D., Kelly, A., Koutsakos, M., Kent, S.J., Juno, J.A., Tan, H.-X., and Wheatley, A.K. (2025). Deconvolution of cargo delivery and immunogenicity following intranasal delivery of mRNA lipid nanoparticle vaccines. Mol. Ther. Nucleic Acids *36*, 102547. https://doi.org/10.1016/j.omtn.2025.102547.

62. Baldeon Vaca, G., Meyer, M., Cadete, A., Hsiao, C.J., Golding, A., Jeon, A., Jacquinet, E., Azcue, E., Guan, C.M., Sanchez-Felix, X., et al. (2023). Intranasal mRNA-LNP vaccination protects hamsters from SARS-CoV-2 infection. Sci. Adv. *9*, eadh1655. https://doi.org/10.1126/sciadv.adh1655.

63. Kiyono, H., and Ernst, P.B. (2025). Nasal vaccines for respiratory infections. Nature *641*, 321–330. https://doi.org/10.1038/s41586-025-08910-6.

64. Maniyamgama, N., Bae, K.H., Chang, Z.W., Lee, J., Ang, M.J.Y., Tan, Y.J., Ng, L.F.P., Renia, L., White, K.P., and Yang, Y.Y. (2025). Muco-penetrating lipid nanoparticles having a liquid core for enhanced intranasal mRNA delivery. Adv. Sci. (Weinh.) *12*, e2407383. https://doi.org/10.1002/advs.202407383.

65. Kent, S.J., Khoury, D.S., Reynaldi, A., Juno, J.A., Wheatley, A.K., Stadler, E., John Wherry, E., Triccas, J., Sasson, S.C., Cromer, D., and Davenport, M.P. (2022). Disentangling the relative importance of T cell responses in COVID-19: leading actors or supporting cast? Nat. Rev. Immunol. *22*, 387–397. https://doi.org/10.1038/s41577-022-00716-1.

66. Liu, J., Yu, J., McMahan, K., Jacob-Dolan, C., He, X., Giffin, V., Wu, C., Sciacca, M., Powers, O., Nampanya, F., et al. (2022). CD8 T cells contribute to vaccine protection against SARS-CoV-2 in macaques. Sci. Immunol. *7*, eabq7647. https://doi.org/10.1126/sciimmunol.abq7647.

67. Moss, P. (2022). The T cell immune response against SARS-CoV-2. Nat. Immunol. *23*, 186–193. https://doi.org/10.1038/s41590-021-01122-w.

68. Duan, S., and Thomas, P.G. (2016). Balancing immune protection and immune pathology by $CD8^+$ T-cell responses to influenza infection. Front. Immunol. *7*, 25. https://doi.org/10.3389/fimmu.2016.00025.

69. Menon, T., Illing, P.T., Chaurasia, P., McQuilten, H.A., Shepherd, C., Rowntree, L. C., Petersen, J., Littler, D.R., Khuu, G., Huang, Z., et al. (2024). CD8+ T-cell responses towards conserved influenza B virus epitopes across anatomical sites and age. Nat. Commun. *15*, 3387. https://doi.org/10.1038/s41467-024-47576-y.

70. Yee, P.T.I., and Poh, C.L. (2018). T cell immunity to Enterovirus 71 infection in humans and implications for vaccine development. Int. J. Med. Sci. *15*, 1143–1152. https://doi.org/10.7150/ijms.26450.

71. Dhalech, A.H., Condotta, S.A., Pattnaik, A., Corn, C., Richer, M.J., and Robinson, C. M. (2023). Coxsackievirus B3 elicits a sex-specific $CD8^+$ T cell response which protects female mice. PLoS Pathog. *19*, e1011465. https://doi.org/10.1371/journal.ppat.1011465.

72. Vecchio, F., Carré, A., Korenkov, D., Zhou, Z., Apaolaza, P., Tuomela, S., Burgos-Morales, O., Snowhite, I., Perez-Hernandez, J., Brandao, B., et al. (2024). Coxsackievirus infection induces direct pancreatic β cell killing but poor antiviral CD8+ T cell responses. Sci. Adv. *10*, eadl1122. https://doi.org/10.1126/sciadv.adl1122.

73. Wu, C.N., Lin, Y.C., Fann, C., Liao, N.S., Shih, S.R., and Ho, M.S. (2001). Protection against lethal enterovirus 71 infection in newborn mice by passive immunization with subunit VP1 vaccines and inactivated virus. Vaccine *20*, 895–904. https://doi.org/10.1016/s0264-410x(01)00385-1.

74. Zhou, S.-L., Ying, X.-L., Han, X., Sun, X.-X., Jin, Q., and Yang, F. (2015). Characterization of the enterovirus 71 VP1 protein as a vaccine candidate: Character of EV 71 VP1 Protein as Vaccine. J. Med. Virol. *87*, 256–262. https://doi.org/10.1002/jmv.24018.

75. Ozdilek, A., and Avci, F.Y. (2022). Glycosylation as a key parameter in the design of nucleic acid vaccines. Curr. Opin. Struct. Biol. *73*, 102348. https://doi.org/10.1016/j.sbi.2022.102348.

76. Rui, Y., Su, J., Wang, H., Chang, J., Wang, S., Zheng, W., Cai, Y., Wei, W., Gordy, J. T., Markham, R., et al. (2017). Disruption of MDA5-mediated innate immune responses by the 3C proteins of Coxsackievirus A16, Coxsackievirus A6, and Enterovirus D68. J. Virol. *91*, e00546-17. https://doi.org/10.1128/JVI.00546-17.

77. Li, X., Guo, H., Yang, J., Liu, X., Li, H., Yang, W., Zhang, L., Li, Y., and Wei, W. (2024). Enterovirus D68 3C protease antagonizes type I interferon signaling by cleaving signal transducer and activator of transcription 1. J. Virol. *98*, e0199423. https://doi.org/10.1128/jvi.01994-23.

78. Zhang, L., Yang, J., Li, H., Zhang, Z., Ji, Z., Zhao, L., and Wei, W. (2023). Enterovirus D68 infection induces TDP-43 cleavage, aggregation, and neurotoxicity. J. Virol. *97*, e0042523. https://doi.org/10.1128/jvi.00425-23.

79. Fujii, K., Nagata, N., Sato, Y., Ong, K.C., Wong, K.T., Yamayoshi, S., Shimanuki, M., Shitara, H., Taya, C., and Koike, S. (2013). Transgenic mouse model for the study of enterovirus 71 neuropathogenesis. Proc. Natl. Acad. Sci. USA *110*, 14753–14758. https://doi.org/10.1073/pnas.1217563110.

80. Zhou, S., Liu, Q., Wu, X., Chen, P., Wu, X., Guo, Y., Liu, S., Liang, Z., Fan, C., and Wang, Y. (2016). A safe and sensitive enterovirus A71 infection model based on human SCARB2 knock-in mice. Vaccine *34*, 2729–2736. https://doi.org/10.1016/j.vaccine.2016.04.029.

81. Piechotta, V., Siemens, W., Thielemann, I., Toews, M., Koch, J., Vygen-Bonnet, S., Kothari, K., Grummich, K., Braun, C., Kapp, P., et al. (2023). Safety and effectiveness of vaccines against COVID-19 in children aged 5-11 years: a systematic review and meta-analysis. Lancet. Child Adolesc. Health *7*, 379–391. https://doi.org/10.1016/S2352-4642(23)00078-0.

82. Copland, E., Patone, M., Saatci, D., Handunnetthi, L., Hirst, J., Hunt, D.P.J., Mills, N. L., Moss, P., Sheikh, A., Coupland, C.A.C., et al. (2024). Safety outcomes following COVID-19 vaccination and infection in 5.1 million children in England. Nat. Commun. *15*, 3822. https://doi.org/10.1038/s41467-024-47745-z.

83. Kärber, G. (1931). Beitrag zur kollektiven Behandlung pharmakologischer Reihenversuche. Archiv f. experiment. Pathol. u. Pharmakol *162*, 480–483. https://doi.org/10.1007/bf01863914.

84. Baiersdörfer, M., Boros, G., Muramatsu, H., Mahiny, A., Vlatkovic, I., Sahin, U., and Karikó, K. (2019). A facile method for the removal of dsRNA contaminant from in vitro-transcribed mRNA. Mol. Ther. Nucleic Acids *15*, 26–35. https://doi.org/10.1016/j.omtn.2019.02.018.

85. Hassett, K.J., Benenato, K.E., Jacquinet, E., Lee, A., Woods, A., Yuzhakov, O., Himansu, S., Deterling, J., Geilich, B.M., Ketova, T., et al. (2019). Optimization of lipid nanoparticles for intramuscular administration of mRNA vaccines. Mol. Ther. Nucleic Acids *15*, 1–11. https://doi.org/10.1016/j.omtn.2019.01.013.

OMTN, Volume *36*

Supplemental information

mRNA vaccine expressing enterovirus D68 virus-like particles induces potent neutralizing antibodies and protects against infection

Yuta Kunishima, Kota Senpuku, Chikako Kataoka-Nakamura, Toshiro Hirai, and Yasuo Yoshioka

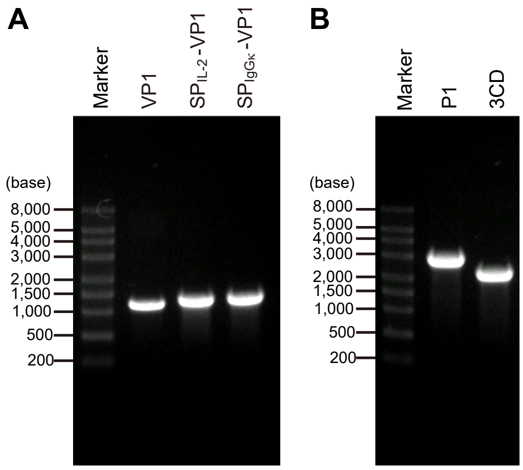

Figure S1. Electrophoresis of mRNA. (A, B) The denatured mRNA samples were electrophoresed in a 1% agarose gel. Marker shows single-stranded RNA ladder. (A) The mRNAs used in Figure 1. (B) The mRNAs used in Figures 2–6.

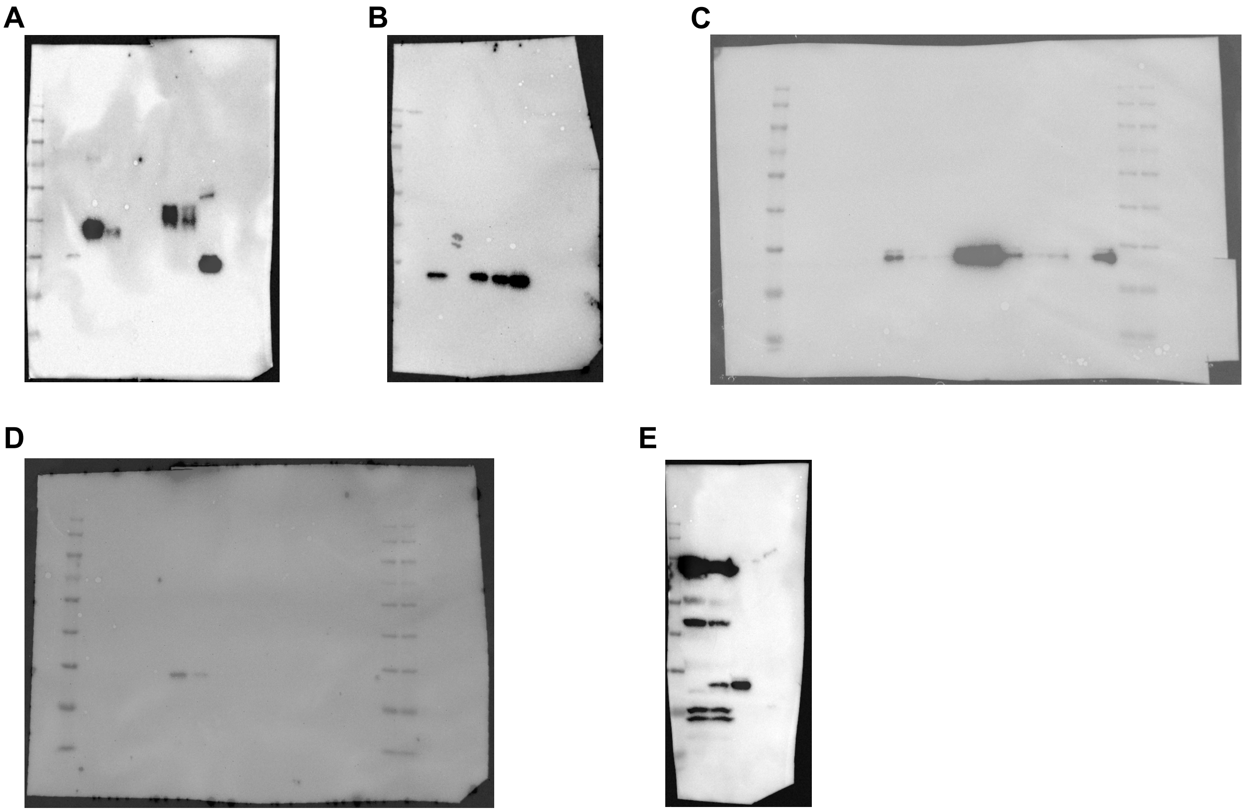

Figure S2. Unedited blotting images. (A) Figure 1B. (B) Figure 2B. （C,D) Figure 2C (E) Figure 2D.

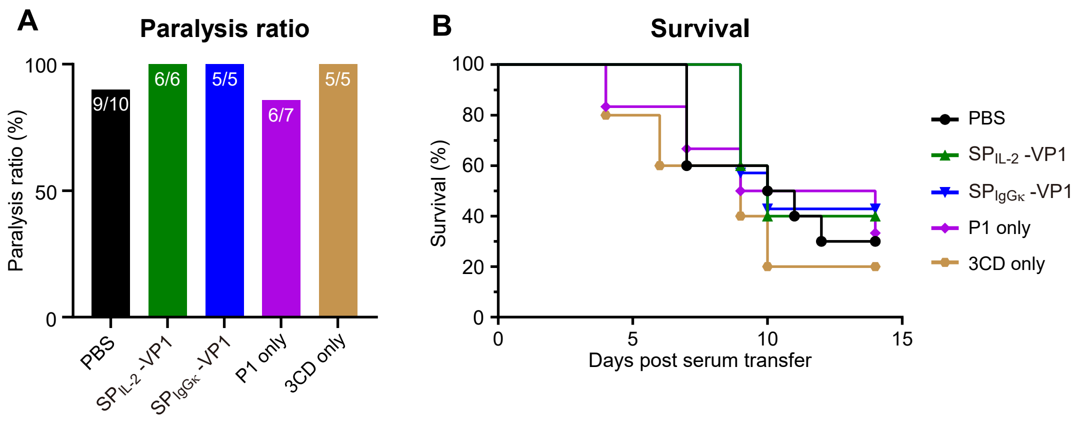

Figure S3. The protective effect of the sera derived from mice vaccinated for VP1, P1, or 3CD. Limb paralysis (A) and rate of survival (B) in the challenged mice were monitored every day for 14 days after the serum transfer. Experiment schema is shown in Figure 3C.

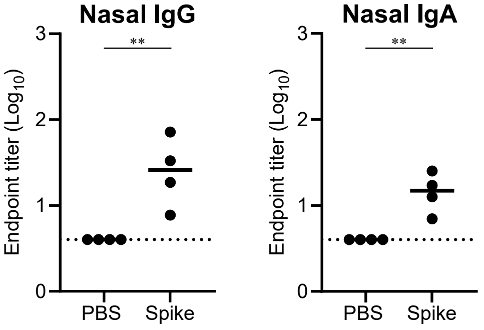

Figure S4. Mucosal antibodies induced by intramuscular vaccination of SARS-CoV-2 mRNA vaccine. Anti-Spike IgG and IgA in nasal wash induced by twice intramuscular immunization of SARS-CoV-2 Spike mRNA-LNP (total 2 μg as mRNA per dose) were quantified with ELISA. Points are presented as individual data and lines are presented as the median. Dotted line represents the detection limit. **, $P < 0.01$ as indicated by Student's *t*-test.

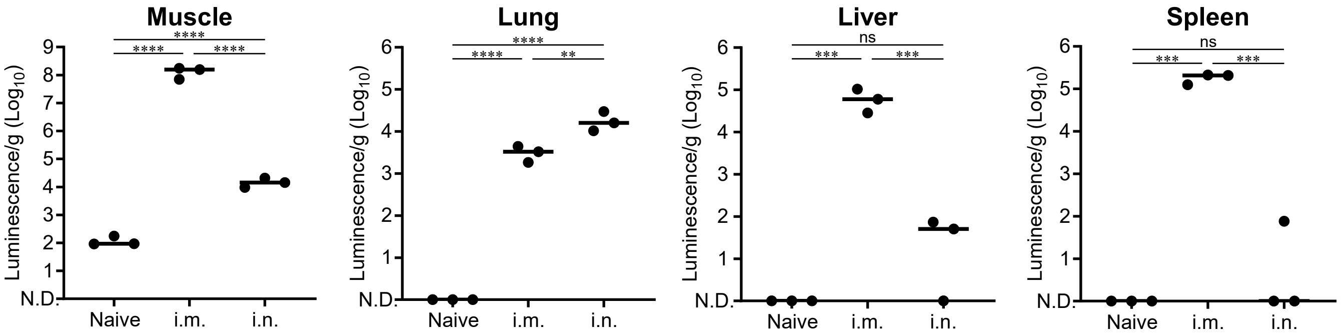

Figure S5. Tissue distribution and expression of mRNA-LNP administrated intramuscularly or intranasally. The mRNA encoding firefly luciferase was encapsulated into LNP and administrated intramuscularly (i.m.) or intranasally (i.n.) to mice (3 μg RNA per dose). The muscle, lung, liver, and spleen were harvested 6 h after administration, and the luciferase activity in the tissue homogenate was quantified. The luminescence was standardized by tissue weight. Points are presented as individual data and lines are presented as median. N.D. means not detectable. **, $P < 0.01$; ***, $P < 0.001$; ****, $P < 0.0001$; ns, not significant as indicated by Tukey's test. (Continued from Fig. 5G.)

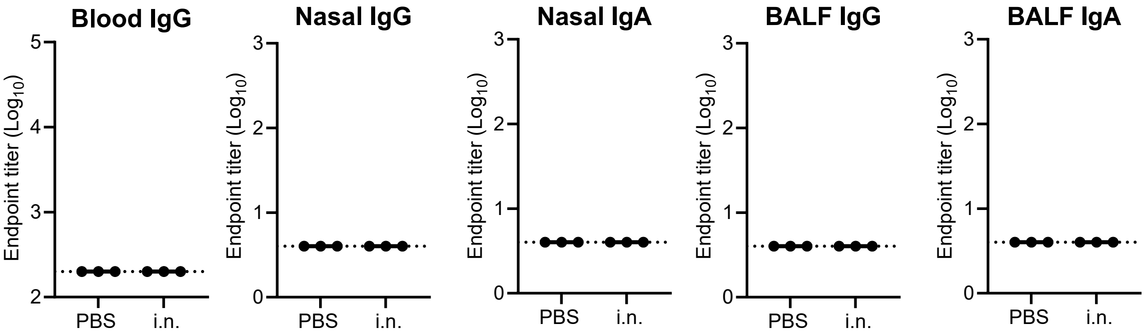

Figure S6. Systemic and mucosal antibodies induced by intranasal vaccination of the mRNA vaccine expressing VLP. Anti-EV-D68 IgG in plasma, IgG and IgA in nasal wash, and IgG and IgA in BALF induced by twice intranasal vaccination of the mRNA vaccine expressing VLP (P1:3CD = 1:1 on weight, total 2 μg as mRNA per dose) were quantified with ELISA. Points are presented as individual data and lines are presented as the median. Dotted line represents the detection limit.

```
EV-D68_MO_3CD       GPGFDFAQAIMKKNTVIARTEKGEFTMLGVYDRVAVIPTHASVGETIYINDVETKVLDAC
CVB3_Nancy_3CD      GPAFEFAVAMMKRNSSTVKTEYGEFTMLGIYDRWAVLPRHAKPGPTILMNDQEVGVLDAK
                    ** * ** * ** *     ** ******* *** ** * **  * **  * *  **** 

EV-D68_MO_3CD       ALRDLTDTNLEITIVKLDRNQKFRDIRHFLPRYEDDYNDAVLSVHTSKFPNMYIPVGQVT
CVB3_Nancy_3CD      ELVDKDGTNLELTLLKLNRNEKFRDIRGFLAKEEVEVNEAVLAINTSKFPNMYIPVGQVT
                     * *   ***** *  ** ** ****** **   *   * ***   ***************

EV-D68_MO_3CD       NYGFLNLGGTPTHRILMYNFPTRAGQCGGVVTTTGKVIGIHVGGNGAQGFAAMLLHSYFS
CVB3_Nancy_3CD      EYGFLNLGGTPTKRMLMYNFPTRAGQCGGVLMSTGKVLGIHVGGNGHQGFSAALLKHYFN
                     *********** * ***************   **** ******** *** * **  ** 

EV-D68_MO_3CD       DTQGEIVSSEKSG----VCINAPAKTKLQPSVFHQVFEGSKEPAVLNPKDPRLKTDFEEA
CVB3_Nancy_3CD      DEQGEIEFIESSKDAGFPVINTPSKTKLEPSVFHQVFEGNKEPAVLRSGDPRLKANFEEA
                    * ****   * *       ** * **** ********* ******   *****  ****

EV-D68_MO_3CD       IFSKYTGNKIMLMDEYMEEAVDHYVGCLEPLDISVDPIPLESAMYGMDGLEALDLTTSAG
CVB3_Nancy_3CD      IFSKYIGNVNTHVDEYMLEAVDHYAGQLATLDISTEPMKLEDAVYGTEGLEALDLTTSAG
                    ***** **     **** ****** * *  ****  *  ** * **  ************

EV-D68_MO_3CD       FPYLLQGKKKRDIFNRHTRDTSEMTKMLEKYGVDLPFVTFVKDELRSREKVEKGKSRLIE
CVB3_Nancy_3CD      YPYVALGIKKRDILSKKTKDLTKLKECMDKYGLNLPMVTYVKDELRSIEKVAKGKSRLIE
                     **   * *****    * *         ***  ** ** ******* *** ********

EV-D68_MO_3CD       ASSLNDSVAMRVAFGNLYATFHNNPGTATGSAVGCDPDIFWSKIPILLDGEIFAFDYTGY
CVB3_Nancy_3CD      ASSLNDSVAMRQTFGNLYKTFHLNPGVVTGSAVGCDPDLFWSKIPVMLDGHLIAFDYSGY
                    ***********  ***** *** ***  ******** * *****  ***   **** **

EV-D68_MO_3CD       DASLSPVWFACLKKVLIKLGYTHQTS-FIDYLCHSVHLYKDKKYIVNGGMPSGSSGTSIF
CVB3_Nancy_3CD      DASLSPVWFACLKMLLEKLGYTHKETNYIDYLCNSHHLYRDKHYFVRGGMPSGCSGTSIF
                    *************  * ******     ***** * ***  ** * * ****** ******

EV-D68_MO_3CD       NTMINNIIIRTLLIRVYKGIDLDQFKMIAYGDDVIASYPHKIDPGLLAEAGKQYGLVMTP
CVB3_Nancy_3CD      NSMINNIIIRTLMLKVYKGIDLDQFRMIAYGDDVIASYPWPIDASLLAEAGKGYGLIMTP
                    * ********** * ********** ***************  **  ******* *** ***

EV-D68_MO_3CD       ADKGTSFIDTNWENVTFLKRYFRADDQYPFLIHPVMPMKEIHESIRWTKDPRNTQDHVRS
CVB3_Nancy_3CD      ADKGECFNEVTWTNATFLKRYFRADEQYPFLVHPVMPMKDIHESIRWTKDPKNTQDHVRS
                    ****  *    * * ********** ***** ******* *********** * ********

EV-D68_MO_3CD       LCYLAWHNGEEAYNEFCRKIRSVPVGRALTLPAYSSLRRKWLDSF
CVB3_Nancy_3CD      LCLLAWHNGEHEYEEFIRKIRSVPVGRCLTLPAFSTLRRKWLDSF
                    ** *******  * ** *********** ***** * *********
```

Figure S7. Protein alignment of the 3CD region of the EV-D68 vaccine strain (MO) with the CVB3 challenge strain (Nancy). Alignment was performed using clastal w. Red charactor shows conserved class I epitope discribed in Discussion section.

```
EV-D68_MO_P1    MGAQVTRQQTGTHENANIATNGSHITYNQINFYKDSYAASASKQDFSQDPSKFTEPVVEG
CVB3_Nancy_P1   MGAQVSTQKTGAHETRLNASGNSIIHYTNINYYKDAASNSANRQDFTQDPGKFTEPVKDI
                *****  * ** **    *   * * *  ** ***    **  *** *** ******

EV-D68_MO_P1    LKAGAPVLKSPSAEACGYSDRVLQLKLGNSAIVTQEAANYCCAYGEWPNYLPDHEAVAID
CVB3_Nancy_P1   MIKSLPALNSPTVEECGYSDRARSITLGNSTITTQECANVVVGYGVWPDYLKDSEATAED
                     * * **  * ******     **** * *** **    ** ** ** * ** * *

EV-D68_MO_P1    KPTQPETATDRFYTLKSVKWETGSTGWWWKLPDALNNIGMFGQNVQHHYLYRSGFLIHVQ
CVB3_Nancy_P1   QPTQPDVATCRFYTLDSVQWQKTSPGWWWKLPDALSNLGLFGQNMQYHYLGRTGYTVHVQ
                 ****  ** ***** ** *   * ********** * * **** * *** * *   ***

EV-D68_MO_P1    CNATKFHQGALLVVAIPEHQRGAHNTNTSPGFDDIMKGEEGGTFNHP-------------
CVB3_Nancy_P1   CNASKFHQGCLLVVCVPEAEMGCATLDNTPSSAELLGGDSAKEFADKPVASGSNKLVQRV
                *** ***** ****  **   *       *       *     *

EV-D68_MO_P1    -YVLDDGTSLACATIFPHQWINLRTNNSATIVLPWMNAAPMDFPLRHNQWTLAIIPVVPL
CVB3_Nancy_P1   VYNAGMGVGVGNLTIFPHQWINLRTNNSATIVMPYTNSVPMDNMFRHNNVTLMVIPFVPL
                 *    *      ******************* *  *  ***   ***  **  ** ***

EV-D68_MO_P1    GTR-TTSSMVPITVSIAPMCCEFNGLRHAITQGVPTYLLPGSGQFLTTDDHSSAPALPCF
CVB3_Nancy_P1   DYCPGSTTYVPITVTIAPMCAEYNGLRLAGHQGLPTMNTPGSCQFLTSDDFQSPSAMPQY
                         ***** ***** * **** *  ** **   *** **** **  *  * *

EV-D68_MO_P1    NPTPEMHIPGQVRNMLEVVQVESMMEINNTESAVG-MERLKVDISALTDVDQLLFNIPLD
CVB3_Nancy_P1   DVTPEMRIPGEVKNLMEIAEVDSVVPVQNVGEKVNSMEAYQIPVRSNEGSGTQVFGFPLQ
                  **** *** * *  *   * *     *    *  **                *  **

EV-D68_MO_P1    IQLDGPLRNTLVGNISRYYTHWSGSLEMTFMFCGSFMAAGKLILCYTPPGGSCPTTRETA
CVB3_Nancy_P1   PGYSSVFSRTLLGEILNYYTHWSGSIKLTFMFCGSAMATGKFLLAYSPPGAGAPTKRVDA
                         ** * *  ********   ******* ** **  * * ***   ** *  *

EV-D68_MO_P1    MLGTHIVWDFGLQSSVTLIIPWISGSHYRMFNNDAKSTNANVGYVTCFMQTNLIVPSESS
CVB3_Nancy_P1   MLGTHVIWDVGLQSSCVLCIPWISQTHYRYVASDEYTAGG---FITCWYQTNIVVPADAQ
                *****  ** *****  * *****  ***    *           **  ***  **

EV-D68_MO_P1    DTCSLIGFIAAKDDFSLRLMRDSPDIGQLDHLHAAEAAYQIESIIKTATDTVKSEINAEL
CVB3_Nancy_P1   SSCYIMCFVSACNDFSVRLLKDTPFISQQNFFQGPVEDAITAAIGRVADTVGTGPTNSEA
                  *    *  *  *** **  * * * *               *   *        * *

EV-D68_MO_P1    GVVPSLNAVETGATSNTEPEEAIQTRTVINQHGVSETLVENFLSRAALVSKRSFEYKDHT
CVB3_Nancy_P1   --IPALTAAETGHTSQVVPGDTMQTRHVKNYHSRSESTIENFLCRSACVYFTEYENS---
                   * * * *** **   *    *** * * *  **   **** * * *     *

EV-D68_MO_P1    SSTARADKNFFKWTINTRSFVQLRRKLELFTYLRFDAEITILTTVAVNGSGNNTYVGLPD
CVB3_Nancy_P1   -----GAKRYAEWVLTPRQAAQLRRKLEFFTYVRFDLELTFVITSTQQPSTTQNQD-AQI
                       *    *    *   ******* *** *** * *   *     *

EV-D68_MO_P1    LTLQAMFVPTGALTPEKQDSFHWQSGSNASVFFKISDPPARITIPFMCINSAYSVFYDGF
CVB3_Nancy_P1   LTHQIMYVPPGGPVPDKVDSYVWQTSTNPSVFWTEGNAPPRMSIPFLSIGNAYSNFYDGW
                ** * * ** *   * * **  **   * ***      * *  ***  *  *** ****

EV-D68_MO_P1    AGFEKNGLYGINPADTIGNLCVRIVNEHQPVGFTVTVRVYMKPKHIKAWAPRPPRTLPYM
CVB3_Nancy_P1   SEFSRNGVYGINTLNNMGTLYARHVNAGSTGPIKSTIRIYFKPKHVKAWIPRPPRLCQYE
                  *  ** ****     * *  * **         * * * **** *** *****   *

EV-D68_MO_P1    SIANANYKGKERAPNALSAIIGNRDSVKTMPHNIVNT
CVB3_Nancy_P1   KAKNVNFQP--------SGVTTTRQSITTMTNTGAF-
                   * *           *     * *  **
```

Figure S8. Protein alignment of the P1 region of the EV-D68 vaccine strain (MO) with the CVB3 challenge strain (Nancy). Alignment was performed using clastal w.

Table S1. The template DNA for *in vitro* transcription used in this study All genes were inserted into the TAKARA IVTpro vector. The sequence of the secretory signaling peptide is underlined.

Gene	Sequence
EV-D68 MO VP1	ATGCTGGATCACCTTCATGCAGCAGAAGCTGCCTACCAGATTGAGTCCATCATCAAGACAGCCACAGA<br>TACCGTGAAATCCGAGATAAACGCTGAACTGGGTGTTGTTCCATCCCTGAATGCAGTAGAAACAGGAG<br>CCACTTCCAATACCGAACCTGAAGAGGCCATTCAGACACGCACCGTGATTAACCAGCATGGCGTAAGC<br>GAAACTCTGGTCGAGAATTTCCTCTCTCGCGCTGCCTTGGTCAGCAAACGCAGCTTCGAATACAAGGA<br>TCACACGTCATCAACCGCTAGAGCGGACAAGAACTTCTTCAAGTGGACTATCAACACACGGTCCTTTGT<br>GCAACTGCGTAGGAAACTGGAGCTCTTCACGTACCTCAGATTTGACGCGGAGATTACCATTCTCACCA<br>CTGTGGCTGTTAACGGCAGTGGAAACAATACTTATGTCGGTCTGCCTGACCTGACCTTGCAGGCAATG<br>TTTGTACCGACAGGAGCCCTTACTCCCGAGAAACAGGACAGCTTTCATTGGCAGTCAGGCAGCAATGC<br>TAGTGTGTTCTTCAAGATCAGTGATCCACCAGCTCGGATTACCATCCCCTTCATGTGCATCAACTCTGC<br>CTATAGCGTTTTCTATGACGGCTTTGCCGGATTTGAGAAGAATGGGCTGTATGGGATTAACCCTGCTGA<br>CACAATTGGCAATCTGTGTGTGCGGATCGTGAATGAGCACCAACCAGTGGGGTTTACAGTGACGGTGC<br>GAGTGTACATGAAGCCCAAACACATCAAGGCATGGGCACCCAGACCTCCTAGGACTCTTCCGTACATG<br>AGCATAGCCAACGCGAACTACAAAGGGAAAGAGCGAGCCCCAAATGCACTGTCTGCCATAATCGGTAA<br>CAGGGATTCTGTCAAGACCATGCCCCACAATATCGTCAACACCTGA
EV-D68 MO VP1<br>with human IL-2<br>secretory signal peptide	ATGTACAGAATGCAGCTCCTGTCCTGCATCGCTCTGTCTCTTGCCCTGGTGACCAACAGCCTGGATCA<br>CCTTCATGCAGCAGAAGCTGCCTACCAGATTGAGTCCATCATCAAGACAGCCACAGATACCGTGAAAT<br>CCGAGATAAACGCTGAACTGGGTGTTGTTCCATCCCTGAATGCAGTAGAAACAGGAGCCACTTCCAAT<br>ACCGAACCTGAAGAGGCCATTCAGACACGCACCGTGATTAACCAGCATGGCGTAAGCGAAACTCTGGT<br>CGAGAATTTCCTCTCTCGCGCTGCCTTGGTCAGCAAACGCAGCTTCGAATACAAGGATCACACGTCAT<br>CAACCGCTAGAGCGGACAAGAACTTCTTCAAGTGGACTATCAACACACGGTCCTTTGTGCAACTGCGT<br>AGGAAACTGGAGCTCTTCACGTACCTCAGATTTGACGCGGAGATTACCATTCTCACCACTGTGGCTGTT<br>AACGGCAGTGGAAACAATACTTATGTCGGTCTGCCTGACCTGACCTTGCAGGCAATGTTTGTACCGAC<br>AGGAGCCCTTACTCCCGAGAAACAGGACAGCTTTCATTGGCAGTCAGGCAGCAATGCTAGTGTGTTCT<br>TCAAGATCAGTGATCCACCAGCTCGGATTACCATCCCCTTCATGTGCATCAACTCTGCCTATAGCGTTT<br>TCTATGACGGCTTTGCCGGATTTGAGAAGAATGGGCTGTATGGGATTAACCCTGCTGACACAATTGGC<br>AATCTGTGTGTGCGGATCGTGAATGAGCACCAACCAGTGGGGTTTACAGTGACGGTGCGAGTGTACAT<br>GAAGCCCAAACACATCAAGGCATGGGCACCCAGACCTCCTAGGACTCTTCCGTACATGAGCATAGCCA<br>ACGCGAACTACAAAGGGAAAGAGCGAGCCCCAAATGCACTGTCTGCCATAATCGGTAACAGGGATTCT<br>GTCAAGACCATGCCCCACAATATCGTCAACACCTGA
EV-D68 MO VP1<br>with human IgG κchain<br>secretory signal peptide	ATGGAAACCGATACCCTCCTCTTGTGGGTCCTTCTGCTTTGGGTGCCTGGGAGCACTGGCGATCTGGA<br>TCACCTTCATGCAGCAGAAGCTGCCTACCAGATTGAGTCCATCATCAAGACAGCCACAGATACCGTGA<br>AATCCGAGATAAACGCTGAACTGGGTGTTGTTCCATCCCTGAATGCAGTAGAAACAGGAGCCACTTCC<br>AATACCGAACCTGAAGAGGCCATTCAGACACGCACCGTGATTAACCAGCATGGCGTAAGCGAAACTCT<br>GGTCGAGAATTTCCTCTCTCGCGCTGCCTTGGTCAGCAAACGCAGCTTCGAATACAAGGATCACACGT<br>CATCAACCGCTAGAGCGGACAAGAACTTCTTCAAGTGGACTATCAACACACGGTCCTTTGTGCAACTG<br>CGTAGGAAACTGGAGCTCTTCACGTACCTCAGATTTGACGCGGAGATTACCATTCTCACCACTGTGGC<br>TGTTAACGGCAGTGGAAACAATACTTATGTCGGTCTGCCTGACCTGACCTTGCAGGCAATGTTTGTACC<br>GACAGGAGCCCTTACTCCCGAGAAACAGGACAGCTTTCATTGGCAGTCAGGCAGCAATGCTAGTGTGT<br>TCTTCAAGATCAGTGATCCACCAGCTCGGATTACCATCCCCTTCATGTGCATCAACTCTGCCTATAGCG<br>TTTTCTATGACGGCTTTGCCGGATTTGAGAAGAATGGGCTGTATGGGATTAACCCTGCTGACACAATTG<br>GCAATCTGTGTGTGCGGATCGTGAATGAGCACCAACCAGTGGGGTTTACAGTGACGGTGCGAGTGTA<br>CATGAAGCCCAAACACATCAAGGCATGGGCACCCAGACCTCCTAGGACTCTTCCGTACATGAGCATAG<br>CCAACGCGAACTACAAAGGGAAAGAGCGAGCCCCAAATGCACTGTCTGCCATAATCGGTAACAGGGA<br>TTCTGTCAAGACCATGCCCCACAATATCGTCAACACCTGA

Table S1. *Continued*

Gene	Sequence
EV-D68 P1	ATGGGCGCACAAGTAACCCGCCAGCAGACTGGTACTCACGAGAACGCAAACATCGCCACTAATGGGT<br>CACACATCACCTATAACCAGATAAATTTCTACAAAGACTCCTATGCTGCATCCGCATCCAAACAAGATTT<br>CTCACAGGATCCCTCTAAGTTCACTGAACCCGTGGTGGAGGGCCTGAAAGCCGGAGCACCAGTGCTT<br>AAGTCCCCATCTGCCGAGGCGTGTGGCTACTCCGACCGAGTATTGCAGCTGAAACTTGGGAATAGTGC<br>CATCGTGACTCAGGAAGCTGCGAATTACTGCTGCGCCTATGGCGAATGGCCCAATTATCTGCCAGATC<br>ATGAGGCAGTCGCTATTGACAAGCCAACACAACCAGAAACCGCAACAGACAGGTTTTACACGCTGAAA<br>TCCGTGAAGTGGGAAACCGGTAGCACAGGATGGTGGTGGAAACTGCCTGATGCACTCAACAACATTG<br>GGATGTTCGGGCAGAATGTGCAGCACCATTACCTGTACAGAAGCGGGTTTCTGATACACGTGCAGTGC<br>AATGCTACAAAGTTTCACCAAGGCGCTCTCCTGGTTGTAGCCATTCCCGAGCATCAGAGAGGAGCACA<br>CAATACTAATACTAGCCCTGGGTTCGACGATATCATGAAAGGGGAGGAAGGAGGCACATTCAATCATC<br>CCTATGTCTTGGATGACGGCACGTCACTTGCGTGTGCTACTATATTTCCACACCAGTGGATAAATCTGC<br>GCACAAACAACAGCGCTACTATTGTGCTGCCCTGGATGAACGCTGCACCCATGGACTTCCCACTGCGG<br>CATAATCAGTGGACTCTGGCCATAATCCCTGTAGTGCCTCTTGGAACTCGTACCACGTCTAGCATGGTT<br>CCCATCACGGTGTCTATTGCGCCGATGTGTTGCGAATTCAATGGCCTGCGACATGCGATTACACAGGG<br>AGTCCCAACCTACCTCCTCCCAGGGAGTGGACAGTTTCTCACTACCGATGACCACAGCAGTGCCCCAG<br>CTCTGCCTTGCTTCAACCCAACTCCGGAGATGCACATTCCTGGGCAAGTGCGCAATATGCTGGAGGTG<br>GTTCAGGTCGAGTCTATGATGGAGATCAACAATACCGAGTCTGCCGTGGGTATGGAGCGACTCAAAGT<br>GGACATCTCCGCTCTGACAGACGTTGACCAACTGCTGTTTAACATTCCCCTCGACATCCAGCTCGATG<br>GACCCTTGCGCAACACCTTGGTGGGGAACATAAGTCGCTACTACACCCATTGGAGCGGGAGCCTGGA<br>AATGACCTTTATGTTCTGTGGGAGCTTTATGGCTGCAGGCAAGCTGATACTGTGCTATACCCCTCCTGG<br>CGGATCATGTCCCACAACCAGGGAGACAGCCATGCTGGGAACCCACATTGTGTGGGACTTTGGTCTG<br>CAATCCTCCGTGACTCTCATCATCCCTTGGATTAGTGGCTCACACTACCGGATGTTTAACAACGATGCC<br>AAATCCACCAACGCTAATGTGGGATATGTGACTTGTTTCATGCAGACTAACCTCATTGTGCCCTCTGAG<br>AGCAGCGATACTTGCTCACTTATCGGCTTCATTGCGGCCAAGGATGACTTTTCTCTTAGGCTGATGCG<br>GGACTCTCCGGATATCGGACAACTGGACCATCTGCATGCTGCCGAGGCCGCTTACCAGATTGAAAGCA<br>TAATCAAGACAGCGACAGACACAGTAAAGTCCGAGATTAACGCAGAGCTGGGTGTTGTGCCCTCACTC<br>AACGCTGTCGAAACAGGAGCCACCTCCAACACAGAACCTGAAGAAGCCATCCAGACCAGAACCGTTAT<br>CAACCAGCATGGCGTGAGCGAGACATTGGTCGAAAACTTTCTGTCCAGGGCCGCATTGGTCAGCAAG<br>CGTAGCTTTGAGTACAAGGATCACACGAGCAGCACAGCTAGAGCCGATAAGAACTTCTTTAAGTGGAC<br>GATTAATACCCGGAGTTTCGTTCAGCTGCGCCGGAAACTGGAACTGTTTACATATCTCAGGTTTGATGC<br>CGAGATTACCATTCTGACCACGGTCGCCGTCAATGGTTCTGGTAATAACACCTATGTGGGGCTTCCTG<br>ACCTGACCTTGCAGGCTATGTTTGTGCCTACTGGCGCACTGACCCCGGAAAAGCAGGACAGTTTCCAT<br>TGGCAGAGTGGCTCTAACGCCAGCGTGTTCTTCAAGATCTCCGACCCTCCCGCTAGAATCACGATCCC<br>ATTCATGTGCATCAACTCAGCCTATTCAGTATTCTATGATGGCTTTGCCGGTTTCGAGAAGAATGGCCT<br>GTACGGAATCAATCCAGCAGATACAATCGGCAATCTCTGTGTCCGTATTGTCAATGAGCACCAACCCG<br>TCGGTTTCACCGTTACAGTGAGGGTTTACATGAAGCCGAAACACATCAAAGCCTGGGCACCCAGACCT<br>CCGCGGACCCTTCCTTATATGTCTATCGCCAACGCTAACTACAAAGGCAAAGAGAGAGCCCCAAATGC<br>CTTGAGTGCCATCATTGGGAATAGGGATAGCGTTAAGACGATGCCCCACAACATAGTCAATACATGA
EV-D68 3CD	ATGGGCCCTGGGTTTGATTTCGCACAAGCCATCATGAAGAAGAACACAGTCATCGCTCGGACAGAGAA<br>GGGGGAGTTCACTATGCTGGGCGTATACGATAGAGTTGCGGTGATTCCCACTCATGCCAGTGTGGGG<br>GAAACGATCTACATCAACGATGTCGAAACAAAGGTGCTGGACGCATGTGCTCTGAGAGACTTAACGGA<br>CACTAACCTGGAGATCACCATTGTGAAGCTGGATCGGAACCAGAAATTTAGAGACATTCGCCATTTCCT<br>GCCCAGGTACGAAGATGACTATAATGACGCTGTATTATCTGTACACACGAGTAAATTCCCGAACATGTA<br>CATTCCCGTTGGCCAAGTCACCAATTACGGGTTTCTTAACCTTGGTGGCACTCCTACCCATAGGATCCT<br>CATGTACAATTTCCCTACACGAGCCGGACAGTGTGGTGGTGTAGTGACAACAACAGGGAAGGTGATTG<br>GGATACACGTCGGTGGTAATGGCGCTCAAGGATTCGCCGCAATGCTCCTTCACTCCTACTTCAGTGAT<br>ACTCAGGGAGAGATCGTTAGCAGCGAAAAGAGCGGAGTGTGCATTAACGCTCCAGCGAAGACCAAAC<br>TCCAGCCTTCTGTGTTTCATCAGGTGTTTGAGGGAAGTAAAGAACCAGCGGTACTAAACCCCAAAGATC<br>CACGCCTCAAGACCGACTTTGAGGAGGCCATTTTCTCCAAATACACGGGAAATAAGATCATGCTGATG<br>GACGAGTACATGGAAGAAGCTGTGGACCACTATGTGGGGTGTCTGGAACCACTCGATATCTCCGTGGA<br>TCCGATCCCACTTGAGTCAGCCATGTACGGTATGGATGGACTTGAAGCACTTGACTTGACCACCTCTG<br>CAGGCTTTCCCTACCTGCTACAGGGGAAGAAAAAAAGGGACATATTCAACCGACATACTAGGGACACC<br>TCTGAGATGACTAAAATGCTGGAGAAATATGGCGTTGATTTACCTTTCGTCACCTTTGTCAAAGATGAG<br>CTGCGATCTCGTGAGAAGGTGGAAAAAGGCAAGAGCCGTTTGATTGAAGCCTCGTCTCTGAACGACTC<br>TGTTGCCATGCGCGTTGCCTTTGGCAATTTGTATGCCACATTTCACAATAATCCTGGCACAGCTACTGG<br>GTCAGCCGTGGGATGTGACCCCGACATCTTCTGGTCCAAAATCCCGATCCTCCTGGATGGCGAGATTT<br>TCGCGTTTGACTATACCGGCTATGATGCTTCCTTGAGCCCTGTGTGGTTCGCATGCTTGAAGAAAGTCC<br>TGATTAAGCTGGGTTACACCCATCAGACTTCCTTTATCGACTACCTCTGCCACAGTGTGCACCTCTACA<br>AGGATAAGAAGTACATCGTGAATGGCGGTATGCCAAGTGGATCCTCAGGAACCTCAATCTTCAACACC<br>ATGATCAACAATATCATCATCCGCACACTGCTGATACGGGTTTATAAGGGCATTGACCTGGATCAGTTC<br>AAGATGATAGCCTATGGAGATGATGTGATAGCCTCGTATCCCCATAAGATAGATCCCGGGTTACTTGCT<br>GAGGCAGGAAAGCAGTATGGGCTCGTCATGACACCTGCCGACAAAGGCACGAGCTTCATTGACACCA<br>ATTGGGAGAATGTGACTTTTCTAAAGCGGTACTTTCGCGCAGACGACCAATACCCCTTCCTAATTCACC<br>CAGTCATGCCAATGAAAGAGATTCACGAAAGCATTCGGTGGACCAAAGACCCCAGAAACACACAGGAT<br>CATGTCAGGAGCCTGTGCTATCTGGCATGGCACAATGGCGAAGAAGCCTATAACGAGTTCTGCCGCAA<br>AATACGGTCAGTGCCAGTTGGGAGAGCCTTGACTCTCCCTGCTTATTCCAGCCTGAGAAGGAAGTGGT<br>TGGACTCATTTTGA
